# Crystal step edges can trap electrons on the surfaces of n-type organic semiconductors

**DOI:** 10.1038/s41467-018-04479-z

**Published:** 2018-05-30

**Authors:** Tao He, Yanfei Wu, Gabriele D’Avino, Elliot Schmidt, Matthias Stolte, Jérôme Cornil, David Beljonne, P. Paul Ruden, Frank Würthner, C. Daniel Frisbie

**Affiliations:** 1 Department of Chemical Engineering and Materials Science, University of Minnesota, Minneapolis, MN 55455 USA; 2 Institut Néel CNRS and Grenoble Alpes University, 25 rue des Martyrs, Grenoble, 38042 France; 3 Institut für Organische Chemie & Center for Nanosystems Chemistry, Universität Würzburg, Am Hubland, Würzburg, 97074 Germany; 4 Service de Chimie des Matériaux Nouveaux, Université de Mons, B-7000 Mons, Belgium; 5 Department of Electrical and Computer Engineering, University of Minnesota, Minneapolis, MN 55455 USA

## Abstract

Understanding relationships between microstructure and electrical transport is an important goal for the materials science of organic semiconductors. Combining high-resolution surface potential mapping by scanning Kelvin probe microscopy (SKPM) with systematic field effect transport measurements, we show that step edges can trap electrons on the surfaces of single crystal organic semiconductors. n-type organic semiconductor crystals exhibiting positive step edge surface potentials display threshold voltages that increase and carrier mobilities that decrease with increasing step density, characteristic of trapping, whereas crystals that do not have positive step edge surface potentials do not have strongly step density dependent transport. A device model and microelectrostatics calculations suggest that trapping can be intrinsic to step edges for crystals of molecules with polar substituents. The results provide a unique example of a specific microstructure–charge trapping relationship and highlight the utility of surface potential imaging in combination with transport measurements as a productive strategy for uncovering microscopic structure–property relationships in organic semiconductors.

## Abstract (web summary)

The microstructure of organic semiconductors affects their transport properties, but directly probing this relationship is challenging. He et al. show that step edges act as electron traps on the surfaces of n-type single crystals, resulting in a field effect transistor mobility that depends on step density.

## Introduction

Discovering the connections between electrical transport properties and the structure of organic semiconductor materials is an overarching goal in organic electronics motivated by the desire to improve material performance^1–3^. In this context, organic semiconductor single crystals play a central role because the spectrum of defect types in these systems is minimized compared to thin films and overall transport performance is correspondingly superior. Indeed, measurements on single crystals have redefined our understanding of the ultimate limits on transport of electrons and holes in organic materials^4–9^. Key observations on benchmark crystals of rubrene, for example, include room temperature field effect hole mobilities^10,11^ above 10 cm^2^ V^−1^ s^−1^, band-like temperature dependence of the mobility (i.e., mobility increasing with cooling down to 100 K)^4^, robust Hall effect behavior^12^, and clear mobility anisotropy correlated with crystal structure^13^. These exciting and exemplary properties inspire current efforts to further improve organic semiconductors for device applications and, from a fundamental standpoint, they support the intriguing physical picture of transport dominated by highly mobile and partially delocalized charge carriers in the crystalline limit where defect densities are low^14–16^.

Yet, frustratingly, most organic semiconductor single crystals do not exhibit outstanding electrical properties^8,9,17–19^. As in thin films, traps for charges can dominate transport resulting in lower room temperature mobilities, increased carrier localization, activated behavior, and the absence of the classical Hall effect^20,21^. While the role of charge traps is broadly appreciated, the microscopic origins of these traps, even in single crystals, remain poorly understood. The absence of appropriate spatially resolved analytical tools—or, more accurately, the paucity of their use—has been a contributing factor to this situation^22,23^.

**Fig. 1:**
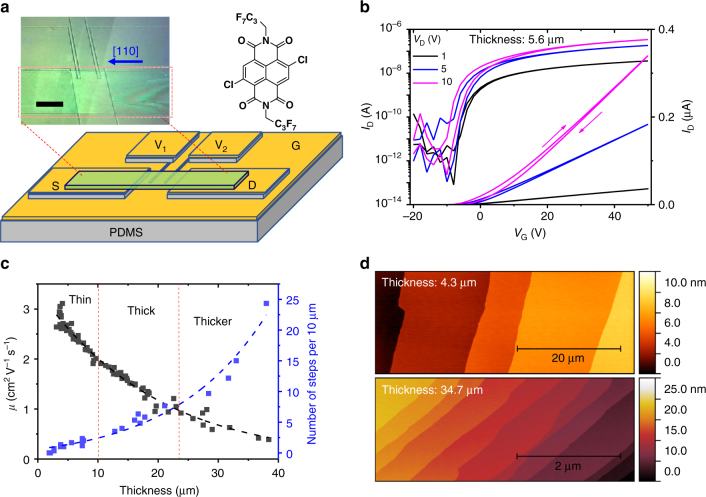
Four-terminal FET measurements for Cl_2_-NDI single crystals with different thicknesses. **a** Schematic diagram of a four-terminal vacuum gap FET configuration on patterned PDMS substrate with a [110]-oriented Cl_2_-NDI single crystal. S, D, G represent source, drain, and gate electrodes, respectively. V_1_ and V_2_ are two voltage sensing channel electrodes. Inset: polarized light microscopy image of a device and chemical structure of Cl_2_-NDI. The black bar scale is 150 µm. **b** Transfer curves (*I*_D_-*V*_G_) for single crystal FETs at different *V*_D_. The extracted electron field effect mobility is ~2.7 cm^2^ V^−1^ s^−1^. The channel length and width are 150 and 200 µm, respectively. The crystal thickness is 5.6 µm. **c** Mobility of 78 crystals (*V*_D_ = 10 V) and step density at the surface of 24 crystals as a function of crystal thickness. The three regions (thin, thick, thicker) correspond to mobility >2 cm^2^ V^−1^ s^−1^ with crystal thickness less than 10 µm, 1–2 cm^2^ V^−1^ s^−1^ with crystal thickness of 10–24 µm and <1 cm^2^ V^−1^ s^−1^with crystal thickness exceeding 24 µm, respectively. The number of steps is measured by atomic force microscopy (AFM) and counted along the direction of the crystal’s long axis ([110] direction). **d** AFM images of step density for single crystals with thicknesses of 4.3 µm (top) and 34.7 µm (bottom), respectively

In this paper, we provide striking evidence that crystal step edges can serve as traps for electrons on the surfaces of n-type organic semiconductor single crystals. Our experimental approach combines quantitative field effect transport measurements as a function of both step density and crystal orientation with scanning probe microscopy, particularly scanning Kelvin probe microscopy (SKPM), which maps surface electrical potentials with sub-50 nm resolution^24–26^. We focus primarily on single crystals of a prototypical n-type organic semiconductor, *N*,*N*’-bis-(heptafluorobutyl)-2,6-dichloro-1,4,5,8-naphthalene tetracarboxylic diimide (Cl_2_-NDI)^18,19,27^, Fig. 1a, that previously has been shown to exhibit outstanding performance in field-effect transistors (FETs). Topographic imaging by atomic force microscopy (AFM) reveals that the density of parallel crystal steps on the (001) major facet increases with crystal thickness and thus controlling thickness by growth conditions provides tunable step density. Qualitatively, we observe that thin Cl_2_-NDI crystals, having fewer steps, display far better transport properties, i.e., higher mobilities and lower threshold voltages. Quantitative FET measurements on the (001) surfaces of ~80 crystals having a range of thicknesses, and thus a spectrum of step densities, show that electron mobility (*μ*) and the device threshold voltage (*V*_T_) strongly decrease and increase, respectively, with increasing step density, which is indicative of charge trapping. Furthermore, there is a pronounced crystallographic anisotropy to these results, namely the largest impact on *μ* and *V*_T_ as step density increases occurs when the transport direction is perpendicular to the step edges; transport parallel to the steps is less affected, which indicates that increased trapping for thicker crystals is predominantly due to step edges and not a homogeneous spatial distribution of traps. As described in detail below, the collective results provide compelling evidence that step edges serve as electron traps on the surfaces of Cl_2_-NDI crystals.

A plausible mechanism for electron trapping at crystal steps is afforded by intriguing high-resolution SKPM images that show the steps have a significant positive electric surface potential relative to the flat terraces. Theoretical consideration of the electrostatics leads to the conclusion that the local positive potential can trap electrons at step edges by creating bound states in the electronic gap. We have developed a device model for FETs with step edge traps that semi-quantitatively accounts for the *V*_T_ shift and provides an estimate of the linear trap density along an edge. We have also confirmed that positive step edge potentials are detected in crystals of other common n-type organic semiconductors, namely fluorocarbon substituted dicyanoperylene-3,4:9,10-bis(dicarboximide) (PDIF-CN_2_)^8^ and 2,5-difluoro-7,7,8,8-tetracyanoquinodimethane (F_2_-TCNQ)^9^, and that the transport properties of these crystals also degrade as step density increases, indicating that our experimental findings have general relevance. Overall, our combined experimental and theoretical analysis indicates that we have discovered a unique microscopic mechanism for electron trapping on the surface of prototypical n-type organic semiconductors, opening up intriguing future opportunities to design materials where such effects are mitigated.

## Results

### FET performance vs. crystal thickness

Lath-like single crystals of *β*-phase Cl_2_-NDI, with lengths up to several mm and thicknesses ranging from a few μm to tens of μm, were grown by physical vapor transport (PVT) in a glass tube subject to a temperature gradient. Thicker crystals grew at zone temperatures near 150 °C while the thinnest crystals grew at temperatures closer to 130 °C. *β*-phase Cl_2_-NDI belongs to the triclinic system, space group P-1 with *a* = 5.1862(4) Å, *b* = 6.3422(5) Å, *c* = 18.4245(15) Å, and *α* = 98.7225(18)°, *β* = 91.6531(19)°, *γ* = 109.6865(18)° at 100 K. The crystals exhibit a two-dimensional brick-wall packing motif in the (001) plane, which constitutes the major facet^19^. Individual single crystals were laminated onto gold-coated poly(dimethylsiloxane) (PDMS) stamps to form four-terminal “vacuum gap” FETs in which the free space between the recessed gate electrode and the crystal serves as the gate insulator, Fig. 1a (see structural characterization in Supplementary Figs. 1–5). This architecture is well known to produce superior FET performance because it minimizes interfacial defects and carrier localization caused by contacting gate dielectric materials directly to the semiconductor^11,28^. Typical transfer curves for a thin crystal at different drain voltages (*V*_D_) are shown in Fig. 1b and are notable in terms of their linearity at high gate voltage (*V*_G_) and relative lack of hysteresis. In total, 78 devices were tested, and we observed a striking inverse correlation between crystal thickness and the room temperature electron field effect mobility, Fig. 1c. Thin crystals with thicknesses below 10 μm exhibited an average electron field effect mobility of ~2.5 ± 0.3 cm^2^ V^−1^ s^−1^, comparable to previous reports^19^, while crystals of intermediate thicknesses, ~10–24 μm, and the highest thicknesses, 24–40 μm, exhibited average mobilities of 1.5 ± 0.2 cm^2^ V^−1^ s^−1^ and 0.7 ± 0.2 cm^2^ V^−1^ s^−1^, respectively. The mobility results suggest that more charge traps exist on the surfaces of thicker crystals^14^. A pronounced drain field (*V*_D_) effect was also found in thick single crystal devices (Supplementary Fig. 6), consistent with a higher degree of charge trapping in thicker crystals^29^.

We characterized the crystals by multiple techniques in an effort to identify the source of charge trapping. Neither optical microscopy nor X-ray diffraction (XRD) revealed any significant differences between thin and thick crystals (see Supplementary Figs. 1–3). The only observable difference in crystal quality was the surface step density on the principal (001) facet measured by AFM, as shown in Fig. 1d and Supplementary Fig. 4. Crystals with thicknesses near 40 μm had more than ten times the number of steps per unit of crystal length (~20 steps/10 μm) than the thinnest crystals with thicknesses of just a few μm (~1 step/10 μm). Such variations in step density with crystal thickness are commonly observed in a variety of materials and reflect changes in step flow growth kinetics with temperature and vapor flux^30–32^. As expected, the step heights were 1.8 nm, corresponding to the *c*-axis dimension of the triclinic Cl_2_-NDI unit cell, i.e., the thickness of one Cl_2_-NDI layer. The inverse correlation between mobility and step density in Fig. 1c strongly suggested that crystal steps were the source of electron traps and lower mobility.

### FET mobility depends on step density and orientation

**Fig. 2:**
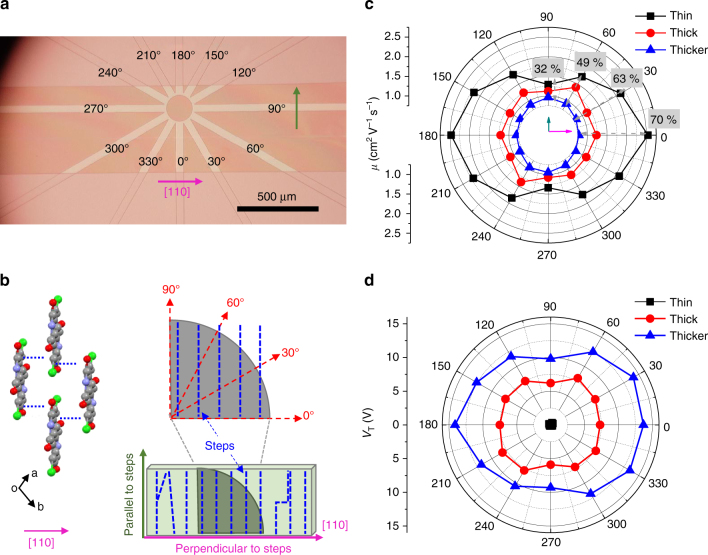
Angle-dependent charge transport at room temperature for Cl_2_-NDI single crystals with different thicknesses. **a** Optical micrograph of Cl_2_-NDI vacuum gap FETs for mobility anisotropy measurement. A fan-shaped contact pattern was employed to probe transport in different angular directions. 0° corresponds to the [110] direction and the crystal long-axis. **b** Crystal packing structure (left, CH_2_C_3_F_7_ omitted for clarity and black dashed lines represent π–π intermolecular interactions) and schematic diagram of crystal steps (blue dashed lines) on the (001) crystal facet (right). Crystal steps are almost perpendicular to the [110] direction. **c**, **d** Mobility and *V*_T_ anisotropy, respectively, for Cl_2_-NDI devices with different crystal thicknesses. Inserted percentages on the mobility polar plot in **c** represent mobility degradation [(*μ*_thin_−*μ*_thicker_)/*μ*_thin_] in the respective directions

To confirm that the degraded mobility in thicker crystals was predominantly due to oriented step edges and not homogeneously distributed traps, we performed FET measurements as a function of crystallographic direction using an array of fan-shaped source and drain contacts, Fig. 2a. Figure 2b indicates that the [110] crystallographic direction, corresponding to the long axis of the crystal, is approximately perpendicular to the step edges. We defined the transport direction in terms of the angle counter-clockwise from [110]. Correspondingly, Fig. 2c is a plot of the two-dimensional electron mobility tensor on the (001) facet for three different Cl_2_-NDI crystal thicknesses; 0˚ is perpendicular to the step edges and 90˚ is parallel. Figure 2d is the corresponding graph for the threshold voltage. For thin crystals the mobility is highest at 0˚ and lowest at 90˚ (black curve), which is expected as 0˚ corresponds to the [110] pi-stacking direction with a high degree of LUMO orbital overlap. However, for the thicker crystal (blue curve), the mobility is substantially lower in the [110] direction, and crucially the angular dependence of the mobility is opposite that for thin crystals, i.e., the mobility is highest at 90˚ parallel to the steps and smallest at 0˚ perpendicular to the steps. Note also that the change in *μ* in the 90˚ direction for thin to thicker crystals is −32%, whereas the change in the 0˚ direction is more than double at −70%. The impact of the steps is clearly greatest in the 0˚ direction. Likewise, Fig. 2d shows that the threshold voltage is much larger for thicker crystals than thin crystals in all directions, consistent with increased trapping, and for thicker crystals the threshold voltage is greatest for transport perpendicular to the steps. Together, Fig. 2c, d provide unambiguous evidence for the critical role of the crystal steps; when the step density is high, transport is more efficient parallel to the steps, whereas when the step density is low, transport is optimized in the pi-stacking direction, as expected. The angular dependences of the mobility and threshold voltage confirm that the oriented steps are substantial impediments to field effect transport on the (001) facet of Cl_2_-NDI.

### Step density and temperature-dependent transport

**Fig. 3:**
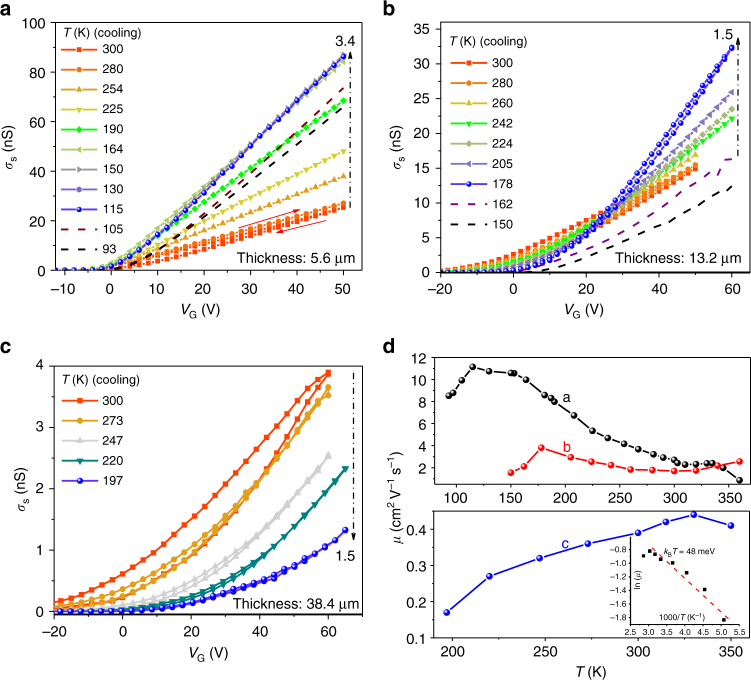
Temperature-dependent FET transport as a function of Cl_2_-NDI crystal thickness. Temperature-dependent *σ*_s_-*V*_G_ characteristics of **a** thin (5.6 μm), **b** intermediate (13.2 μm), and **c** thick (38.4 μm) crystals. The curves change quantitatively and qualitatively: sheet conductance decreases, the temperature dependence inverts, *V*_T_ increases, and hysteresis increases as crystals become thicker. **d** Extracted FET mobility vs. temperature for crystals in **a**–**c**. A clear cross-over from band-like to activated transport is observed. Inset: ln(*μ*) vs. 1000/*T* for the thick crystal data, where the fitting line represents Arrhenius dependence with an activation energy of 48 meV

Trapping effects should manifest themselves in the temperature dependence of FET transport. Accordingly, we carried out temperature-dependent FET measurements in the [110] direction on thin, intermediate, and thick crystals. Figure 3a–c shows the sheet conductance (*σ*_s_)-*V*_G_ characteristics for crystals with thicknesses of 5.6, 13.2, and 38.4 μm, respectively. It is clear that not only does *σ*_s_ at a given *V*_G_ decrease as crystal thickness increases, but also the temperature dependence changes drastically. The transport along [110] is band-like and hysteresis-free for thin crystals (Fig. 3a) and it is activated and hysteretic for thick crystals (Fig. 3c). Correspondingly, in the extracted mobility vs. temperature plots in Fig. 3d, mobility increases with decreasing temperature for the thinnest crystal, reaching 11 cm^2^ V^−1^ s^−1^; this is typical of band-like behavior^4,8,9^. Mobility is essentially flat vs. temperature for the intermediate crystal, and it is activated for the thickest crystal. The associated FET characteristics are shown in Supplementary Figs. 7–9 and Supplementary Table 1. The extracted activation energy for the mobility for thick crystals is 40–80 meV. We have also examined the crystallographic direction dependence and temperature behavior for a relatively thick (34.6 μm) crystal (Supplementary Fig. 10). We find that transport is activated in all directions for this crystal, but the threshold voltage *V*_T_ and activation energy for the mobility are lowest for conduction parallel to the steps and highest along [110] perpendicular to the steps, consistent with expectations based on Figs. 2, 3. Altogether, the data in Figs. 1–3 and Supplementary Figs. 6–10 provide self-consistent evidence that step edges on the (001) face of Cl_2_-NDI crystals serve as electron traps.

### Step edge potential and electron trapping

**Fig. 4:**
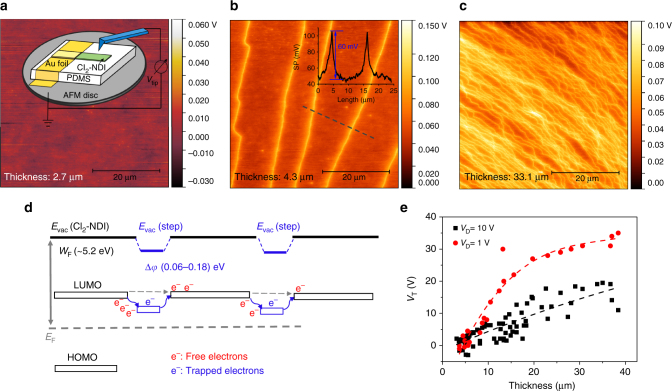
Surface potential maps of crystal step edges and their role as electron traps. **a**–**c** SKPM surface potential images of Cl_2_-NDI single crystals with different thicknesses. Inset to **a**: schematic diagram of the SKPM measurement. Surface potentials averaging +60 mV were observed at discrete step edges in **b**. Inset to **b**: corresponding surface potential profile along the gray dashed line. The thicknesses of crystals were 2.7, 4.3, and 33.1 µm for **a**, **b**, **c**, respectively. **d** The proposed energy level diagram for step edges on the surface of Cl_2_-NDI single crystals. *E*_F_ is the Fermi level and *E*_vac_ is vacuum level. The work function of Cl_2_-NDI single crystal is ~5.2 eV. At the step edge, the vacuum level shifts about 0.06–0.18 eV compared to the surrounding surface by SKPM measurement. **e** The threshold voltage as a function of crystal thickness at *V*_D_ of 10 V (74 devices) and 1 V (25 devices), respectively. The increase in *V*_T_ with crystal thickness is indicative of increased electron trapping

A critical question is the mechanism by which the Cl_2_-NDI crystal step edges trap electrons. Supposing that the crystal steps could have distinct surface potentials, we undertook characterization by SKPM, which has been employed previously to image surface potential domains in organic semiconductors associated with different epitaxial growth modes^33^, residual strain^34^, and grain boundaries^35^. Indeed, as shown in Fig. 4, Cl_2_-NDI step edges have a distinctly more positive surface potential than the (001) terraces. As evident in the line scans, typical step edge potentials were +60 mV, more than twice the thermal voltage *k*_B_*T*/*e* = 25 mV at room temperature and remarkably comparable to the mobility activation energies gleaned from FET measurements. Intersecting step edges have even higher surface potentials, up to approx. +180 mV, as indicated in Fig. 4c and in Supplementary Fig. 5. Step edge potentials are well known in cleaved silicon^36^ and graphite^37^, and were first observed in an organic semiconductor film by Yamagishi et al.^38^. To our knowledge, the data presented in Fig. 4 represent the first detection of step edge potentials in organic semiconductor single crystals.

Positive potentials mean that electron energies are stabilized, and thus we propose that the positive step edge potentials create localized in-gap states, Fig. 4d, that trap electrons. Indeed, as shown in Fig. 4e, we found a systematic positive shift in the *V*_T_ of Cl_2_-NDI FETs as crystal thickness and the step density increased. Figure 4e also shows that larger *V*_D_ values lead to smaller increases in *V*_T_ with thickness, presumably because the lateral field associated with higher *V*_D_ allows more effective release of trapped electrons from step edges^29^.

We have developed a device model of the free carrier concentration in the Cl_2_-NDI crystals in order to estimate the linear trap concentration on the step edges and to predict the variation in *V*_T_ with crystal thickness shown in Fig. 4e. To begin, we assume that there are surface-localized traps on both the top and bottom surfaces that deplete the lightly *n*-doped Cl_2_-NDI (see Supplementary Discussion 1 for the full model and calculation of the equilibrium, ungated carrier concentration). The electrostatic potential energy drop Δ*U* between the bottom crystal surface and the gate electrode in the FET geometry (see Fig. 1a) is then

1 $$\Delta U = \frac{{e^2}}{C}[n_0\left( {d - d_{{\mathrm{dt}}}} \right) - n_{\mathrm{s}} - N_{\mathrm{t}}],$$

where *e* is the fundamental charge, *C* is the specific gate capacitance, *n*_o_ is the equilibrium volumetric carrier concentration, *d* and *d*_dt_ are the crystal thickness and top surface depletion layer thickness, respectively, *n*_s_ is the sheet concentration of free electrons, and *N*_t_ is the average sheet concentration of populated surface traps (to be associated with step edges). Thus, the bracketed terms in Eq. 1 account for all sources of charge, namely ionized donors *n*_0_(*d* − *d*_dt_), free electrons (*n*_s_), and trapped electrons (*N*_t_). (The positive space charge of the top surface depletion layer is neutralized by the negative surface charge of the top surface traps). For large positive gate bias one finds, $\Delta U \approx - eV_{\mathrm{G}}$, where *V*_G_ is the applied gate voltage. Rearranging Eq. 1 then yields,

2 $$n_{\mathrm{s}} \approx n_0\left( {d - d_{{\mathrm{dt}}}} \right) - N_{\mathrm{t}} + (\frac{C}{e})V_{\mathrm{G}}.$$

The mobile sheet carrier concentration *n*_s_ is readily determined from the measured FET drain current at a given *V*_G_ and small *V*_D_. Comparing two FETs based on crystals of different thicknesses *d*_1_ and *d*_2_, but with the same applied gate voltages, we find from Eq. 2 that the difference in their sheet electron concentrations is given by,

3 $$n_{{\mathrm{s}}2} - n_{{\mathrm{s}}1} = n_0\left( {d_2 - d_1} \right) - 2N_{{\mathrm{t}}2} + 2N_{{\mathrm{t}}1},$$

where we have expressed the top surface depletion layer thickness in terms of the corresponding surface trap density, $n_0d_{{\mathrm{dt}}} = N_{\mathrm{t}}$, and we have assumed that top and bottom surfaces have equal trap densities for crystals of a given thickness.

We associate the surface traps on the Cl_2_-NDI crystals with steps between terraces. Hence, we can relate the average surface density of traps *N*_t_ to the linear densities of traps along a step and the linear density of steps across the surface. Both of these quantities are simply expressed in terms of the mean distances between traps along a step and between steps,

4 $$N_{\mathrm{t}} = \frac{1}{{{\mathrm{\Delta }}x{\mathrm{\Delta }}y}}.$$

Here we define the *x*-direction as perpendicular to the steps and the *y*-direction as parallel to the steps. AFM measurements yield 1/Δ*x*, which was found to be approximately proportional to the crystal thickness, $\frac{1}{{{\mathrm{\Delta }}x}} = \alpha d$. We therefore conclude that:

5 $$n_{{\mathrm{s}}2} - n_{{\mathrm{s}}1} = \left( {n_0 - 2\frac{\alpha }{{{\mathrm{\Delta }}y}}} \right)\left( {d_2 - d_1} \right).$$

Taking as a specific example crystals of thickness *d*_1_ = 5.6 μm and *d*_2_ = 24 μm with *V*_G_ = 40 V and the experimentally determined parameters *n*_0_ = 3.5 × 10^13^ cm^−3^ (see Supplementary Discussion 1) and *α* = 3.7 × 10^6^ cm^−2^ (from Fig. 1c) allows us to determine the mean distance between filled traps along a step as Δ*y* = 1.3 nm. That surprisingly small value is not much larger than the lattice constants of Cl_2_-NDI, i.e., there is a filled trap associated with nearly every molecule along a step. We take this as an indication that the traps are probably an intrinsic property of the steps, rather than an effect only associated with surface contamination or aggregation of foreign molecules at the steps. However, more work is required to verify this hypothesis, a point we return to below.

We may also use Eq. 5 to estimate the variation of the threshold voltage with crystal thickness. We find: $\frac{{\partial V_{\mathrm{T}}}}{{\partial d}} \approx - \left( {\frac{e}{C}} \right)\frac{{n_{{\mathrm{s}}2} - n_{{\mathrm{s}}1}}}{{d_2 - d_1}} = - \left( {\frac{e}{C}} \right)\left( {n_0 - 2\frac{\alpha }{{\Delta y}}} \right) = 1.9\,\times10^4\,{\mathrm{V}}\,{\mathrm{cm}}^{ - 1}$. This value is in close agreement with the experimental result (1.7 × 10^4^ V cm^−1^) in Fig. 4e for small *V*_D_ and *d* between 2 and 20 μm. We thus feel the model captures key features of the experimental data and provides a reasonable estimate of the trap density as described above.

### Generality of step edge potential

**Fig. 5:**
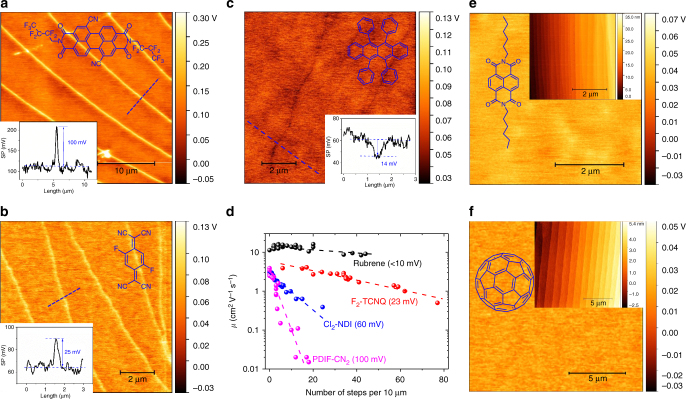
Surface potential maps of other organic crystals and mobility dependence on step density. SKPM surface potential images of n-type PDIF-CN_2_ (**a**), n-type F_2_-TCNQ (**b**), and p-type rubrene (**c**) single crystals. Insets: corresponding molecular structures and surface potential profiles along the blue dashed lines for PDIF-CN_2_, F_2_-TCNQ, and rubrene, respectively. Note that step edge potentials for rubrene, a p-type semiconductor, are negative and weak. **d** Mobility as a function of step density. The black, red, blue, and purple spheres represent rubrene, F_2_-TCNQ, Cl_2_-NDI, and PDIF-CN_2_, respectively. Dashed lines are linear fits. The values in parenthesis are average step edge potentials, and it is clear that the slopes of these plots vary systematically with the magnitude of the step edge potential. SKPM surface potential images of n-type C_6_-NDI (**e**) and n-type C_60_ (**f**) single crystals show no detectable step edge potentials. Insets show the molecular structures and the corresponding AFM height images revealing large numbers of steps

Importantly, the generality of our experimental results are confirmed by examination of FET transport and surface potential images of crystals of other benchmark n-type semiconductors, PDIF-CN_2_^8^ and F_2_-TCNQ^9^. As shown in Fig. 5 and Supplementary Figs. 13–16, crystals of these materials behave similarly to Cl_2_-NDI, namely they also exhibit positive step potentials and mobilities that decrease (and threshold voltages that increase) with increasing step density. In particular, the data in Fig. 5d reveal an especially critical point, namely that the slope of *μ* vs. step density correlates with the average value of the step edge potential: that is, PDIF-CN_2_ has the steepest dependence of *μ* on step density and the largest average step edge potential (+100 mV) and F_2_-TCNQ has the shallowest *μ* vs. step density behavior and the smallest step edge potential (+23 mV). Cl_2_-NDI falls in between on the Fig. 5d plot, consistent with its intermediate +60 mV step edge potential. These data indicate that indeed the electron trapping is strongly correlated with the magnitude of the step edge potential.

Further evidence for this correlation is provided by transport and SKPM data for crystals of three other organic semiconductors, namely p-type rubrene, n-type dihexyl naphthalene diimide (C_6_-NDI)^39^, and n-type C_60_^17^, which all have small or undetectable step edge potentials, Fig. 5c, e, f. For rubrene, Fig. 5c, the edge potentials are slightly negative, which is opposite in sign to edge potentials on n-type Cl_2_-NDI, PDIF-CN_2_, and F_2_-TCNQ. Thus, step edges in rubrene could be anticipated to be traps for holes (the majority carrier), but the magnitude of the potential is small (>−10 mV) and correspondingly the mobility vs. step density relationships are weak for rubrene, as shown explicitly in Fig. 5d (see also Supplementary Fig. 17). For C_6_-NDI and C_60_ crystals, Fig. 5e, f, we found no evidence of step edge potentials, and correspondingly there was no strong dependence of FET mobility on step density (see also Supplementary Fig. 18).

Figure 5 allows us to make several important conclusions: (1) step edge potentials are an important phenomenon in other materials besides Cl_2_-NDI studied extensively here, (2) the magnitude of the step edge potential is correlated with the sensitivity of *μ* to the number of step edges, (3) not all organic semiconductors exhibit step edge potentials, and (4) at least in the cases we have examined so far, when step edge potentials are absent, *μ* does not appear to be sensitive to the density of step edges. In sum then, our results point to the central role of positive step edge potentials for electron trapping on the surface of n-type organic semiconductors.

### Possible origins of step edge potential

Comparison of the molecular structures in Fig. 1a,  5 reveals that only the molecules with strong electron-withdrawing substituents (Cl_2_-NDI, PDIF-CN_2_, and F_2_-TCNQ) exhibit both step edge potentials and the associated dependence of FET transport on step edge density. We have performed microelectrostatics calculations that indicate that for a layer of such molecules, a step edge indeed produces an effective edge dipole, as illustrated in Supplementary Fig. 19 and 20 (see Supplementary Table 2 and Supplementary Discussion 2)^40–42^. The edge dipole in turn produces a large electrostatic potential, Supplementary Figs. 21–24, which could be the source of bound trap states at the edge. The concept of intrinsic step edge potentials for crystals of molecules with electron-withdrawing substituents aligns well with our SKPM data in Fig. 5 and with the high linear density of traps we estimated from the transport measurements. Yet while calculations suggest an intrinsic origin to the edge potential, we anticipate that other factors may also be in play, including, for example, crystallographic reconstruction at the edge^43^ or local contamination^44,45^. Indeed, we have preliminary data that show increased edge potentials upon aging the crystals in air (Supplementary Figs. 25–30). It seems that both intrinsic and extrinsic factors may contribute, and further work will be required to fully elucidate the origins of the step edge potential.

In summary, we have demonstrated the existence of crystal step edge potentials on the surfaces of n-type organic semiconductor single crystals for the first time. We have proposed a plausible mechanism by which these electrically active defects can serve as electron traps, and with complementary transport measurements and a device model we have shown that the density of step edges and the magnitudes of the associated potentials indeed correlate with degraded transport properties in FETs. These findings strongly indicate that step edges can trap electrons on the surfaces of n-type organic semiconductors and provide perhaps the clearest link to date between a specific microstructural feature and charge trapping in an organic material. The results appear to be general, and the spectrum of possible factors contributing to the step edge potential opens up intriguing new directions of inquiry. Our observations that some crystals based on molecules with less polar substituents show much smaller step edge potentials, or no detectable potential at all, may help provide answers in future work.

## Methods

### Crystal growth

Single crystals of *β*-phase Cl_2_-NDI were grown by PVT in a horizontal oven with argon as the inert carrier gas. A temperature gradient was applied, resulting in the evaporation of Cl_2_-NDI at 180 °C at the source and crystallization between 150 and 130 °C. Rubrene^4,10^, PDIF-CN_2_^8^, F_2_-TCNQ^9^, C_6_-NDI^39^, and C_60_^46^ crystals were grown by PVT according to the literature. The thickness of crystals was measured by surface profilometry (KLA-Tencor P-16 surface profiler).

### Four-terminal FET fabrication

3 nm Cr and 20 nm Au were sequentially deposited onto a patterned PDMS substrate, as described previously, to make a four-terminal FET structure with a recessed gate. The distance between source and drain electrodes was 150 µm and the distance between the two channel electrodes (V_1_ and V_2_) was 50 µm. Single crystals of Cl_2_-NDI were laminated across the source, drain, and channel electrodes with the [110] direction (crystal long-axis) parallel to the channel direction. The gate-to-crystal gap is 5 μm in depth for all devices. The specific capacitance is 0.18 nF/cm^2^. The devices were fabricated under ambient conditions and then transferred into an N_2_-filled glove box for measurement. Variable temperature FET measurements were carried out in the dark with a cryogenic probe station at 10^−4^ Torr.

### Scanning Kelvin probe microscopy

Measurements were performed with a Bruker Instruments Nanoscope V Multimode AFM with probes from Mikromasch USA (NSC18, Pt coated, resonant frequency 60–90 kHz, *k* = 2–5.5 N/m, *R*_C_ = 25 nm). All SKPM scans were conducted with AC voltage *V*_AC_ = 6 V and lift height *d* = 10 nm inside an argon-filled glovebox with oxygen levels (∼1 ppm) to mitigate surface contamination effects. The work function of Cl_2_-NDI was referenced to a known standard consisting of a self-assembled monolayer on gold. SKPM measurements were generally performed on the same crystals that were employed in FET measurements. For thin crystals, it was found that the upper and lower (001) faces were morphologically very similar and so in general SKPM was performed on the top surface of the crystal while FET measurements employed the lower surface (opposite face). For the thick single crystals, the two faces sometimes exhibited different morphologies. Thus, the thick crystals were peeled off the patterned PDMS substrates after FET measurements and transferred to the SKPM apparatus so that the same face used in transport could be imaged.

### X-ray analysis

High-resolution XRD was carried out with a Philips Panalytical X’Pert Pro diffractometer with monochromatic Cu Kα radiation (wavelength 0.154 nm) at tube settings of 45 kV and 40 mA.

X-ray photoelectron spectra (XPS) were taken on an SSX-100 XPS (10^−8^ Torr) with an Al Kα X-ray monochromatic source (1486.3 eV) and a hemispherical analyzer. The X-ray anode was operated at 200 W, and the analyzer was set to a pass energy of 150 eV for survey scans and 50 eV for high-resolution scans.

### Data availability

The data that support the findings of this study are available from the corresponding author upon reasonable request.

## Electronic supplementary material

Supplementary Information
