# Supplementary material for: Crystal step edges can trap electrons on the surfaces of n-type organic semiconductors
Source: Nat Commun. 2018 May 30;9:2141. doi: 10.1038/s41467-018-04479-z (PMC5976653; doi:10.1038/s41467-018-04479-z)
Supplement: Supplementary file 1 — Supplementary Information [file 41467_2018_4479_MOESM1_ESM.docx]

**SUPPLEMENTARY INFORMATION**

**Crystal Step Edges Can Trap Electrons on the Surfaces of**

***n*-Type Organic Semiconductors**

Tao He^1^, Yanfei Wu^1^, Gabriele D'Avino^2^, Elliot Schmidt^1^, Matthias Stolte^3^, Jérôme Cornil^4^, David Beljonne^4^, P. Paul Ruden^5^, Frank Würthner^3^, C Daniel Frisbie^1^*

^1^ Department of Chemical Engineering and Materials Science, University of Minnesota,

Minneapolis, Minnesota 55455, USA

^2^ Institut Néel CNRS and Grenoble Alpes University, 25 rue des Martyrs, 38042 Grenoble, France

^3^ Universität Würzburg, Institut für Organische Chemie & Center for Nanosystems Chemistry, Am Hubland, 97074 Würzburg, Germany

^4^ Service de Chimie des Matériaux Nouveaux, Université de Mons, B-7000 Mons, Belgium

^5^Department of Electrical and Computer Engineering, University of Minnesota,

Minneapolis, Minnesota 55455, USA

* Correspondence should be addressed to C Daniel Frisbie

(E-mail: frisbie@umn.edu)


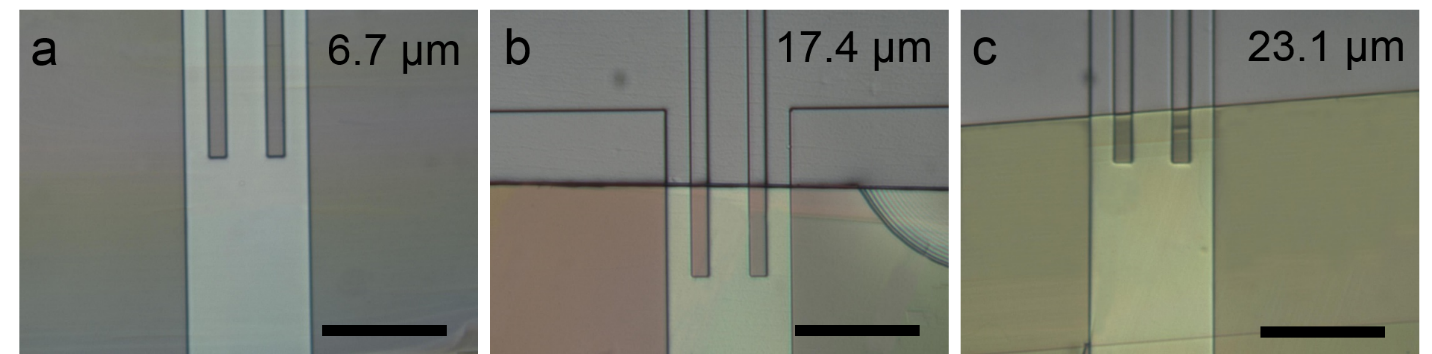


**Supplementary Figure 1 | Optical images of Cl_2_-NDI single crystals with different thickness. a**, 6.7 µm. **b**, 17.4 µm. **c**, 23.1 µm. Crystals with different thicknesses were obtained by controlling the growth period at the same sublimation temperature of 180º C. Usually, thin crystals with a thickness of less than 10 µm could be grown in 5-7 hours. 3-5 days were needed for crystals with a thickness of more than 20 µm. In one batch of crystals, different thicknesses can also be obtained in different deposition zones. The closer to the sublimation zone, the thicker the crystals will be, and the color of crystals will deepen from colorless to yellow with increasing thickness, which can be clearly observed in the channel of devices.


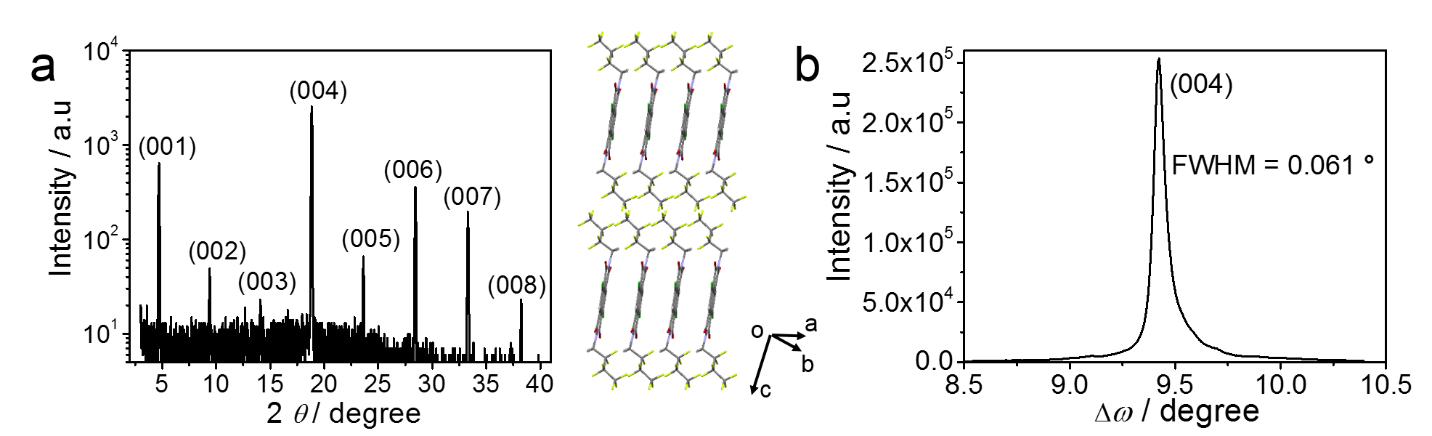


**Supplementary Figure 2 | Wide Angle X-ray diffraction for a Cl_2_-NDI crystal with a thickness of ~ 36 µm. a**, Expected diffraction peaks (2*θ*) of 4.7º, 9.4º, 14.1º, 18.8º, 23.6º, 28.3º, 33.0º, 37.6º (left), corresponding to molecular layer spacing of 18.80 Å, which matches well with the *c*-axis parameters (18.73 Å, 200 K) (right)^1^. **b**, Rocking curve for the (004) plane with a full width at half maximum (FWHM) of 0.061º.


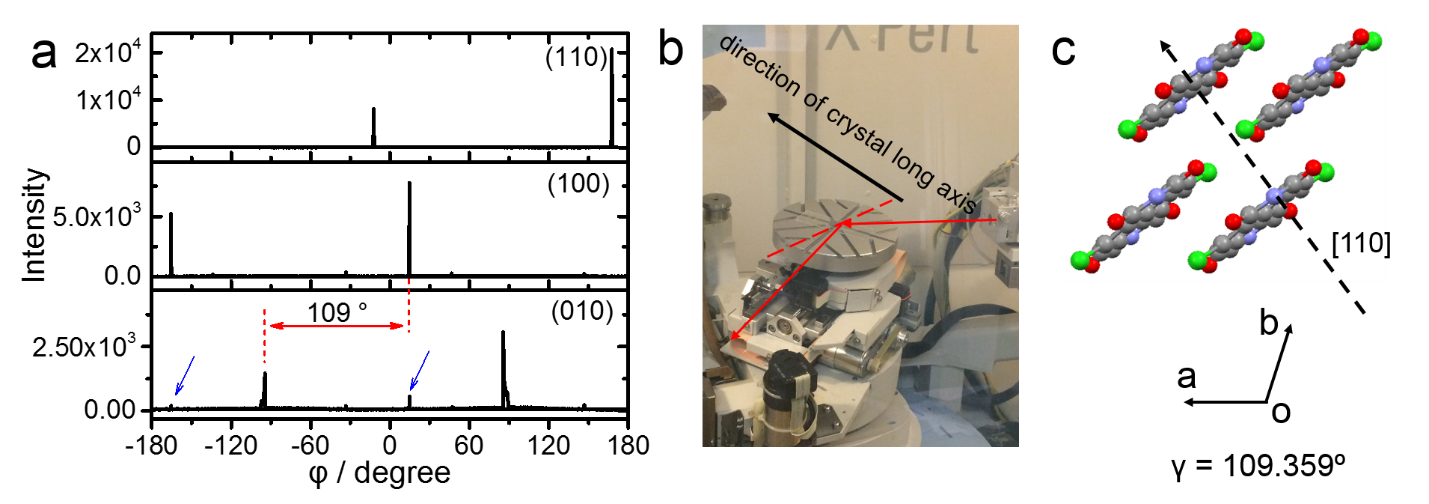


**Supplementary Figure 3 | Grazing incidence X-ray diffraction (GIXD) of a Cl_2_-NDI single crystal.** **a**, In-plane φ scan with 2*θ* fixed at Bragg angles of 27.25º,17.94º and 15.08º for the (110), (100) and (010) planes, respectively. The peak spacings are as expected for the β-phase of Cl_2_-NDI and indicate that the specimen (in the X-ray spot) is a single crystal. Due to very similar 2*θ* angles, the diffraction peaks of (100) plane (blue arrows) can be clearly observed in the *φ* scan of the (010) plane, and are separated from the (010) peaks by *φ* = 109º. That value agrees well with the γ angle of the crystal parameter. **b**, Crystal orientation on the XRD goniometer when the (110) diffraction peak is observed. [110] coincides with the crystal’s long-axis. **c**, Molecular packing structure in the *ab* plane. The [110] orientation is the *π*-stacking direction, which is in good agreement with the principle of optimal crystal growth. The direction of fastest crystalline growth usually corresponds to that with the strongest molecular interaction.


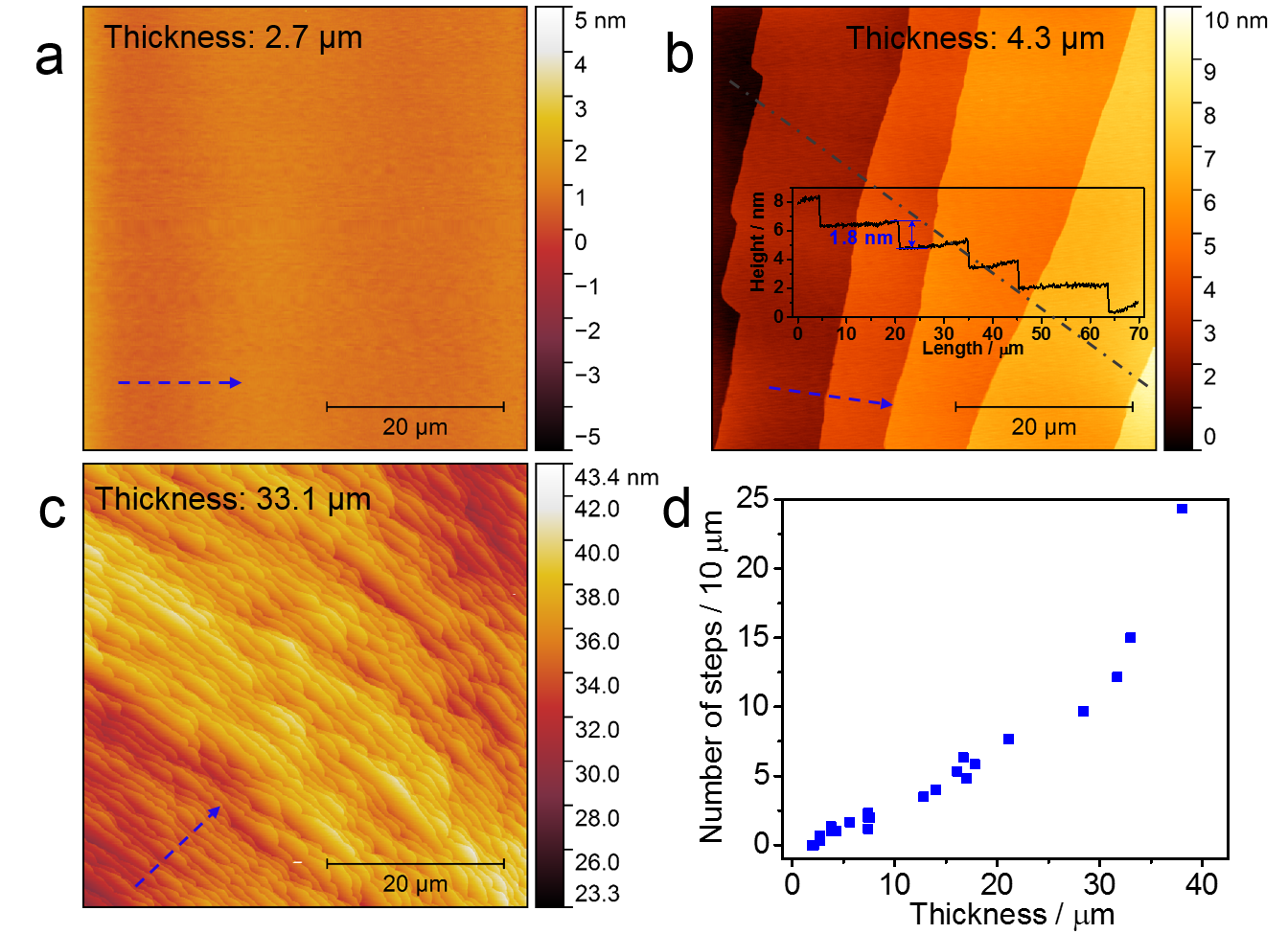


**Supplementary Figure 4 | AFM topographic images of Cl_2_-NDI crystals with different thicknesses. a**, No step in the field of view for a thin crystal with a thickness of 2.7 µm. **b**, Low step density in a crystal with a thickness of 4.3 µm. Terrace profile with a step height of 1.8 nm was obtained, which matches well with the molecular length of 1.85 nm, implying that the molecules nearly stand upright on the surface of the crystal. Closely packed fluoroalkyl-chains lie out of the (001) plane, which leads to a hydrophobic surface and effectively prevents water and oxygen penetration. **c**, High step density in a thick crystal with a thickness of 33.1 µm. **d**, Step density as a function of crystal thickness based on 24 crystals. The number of steps is counted along the direction of the crystal’s long-axis. The blue dashed arrows represent the crystals’ long-axis, which is almost perpendicular to the crystal steps.


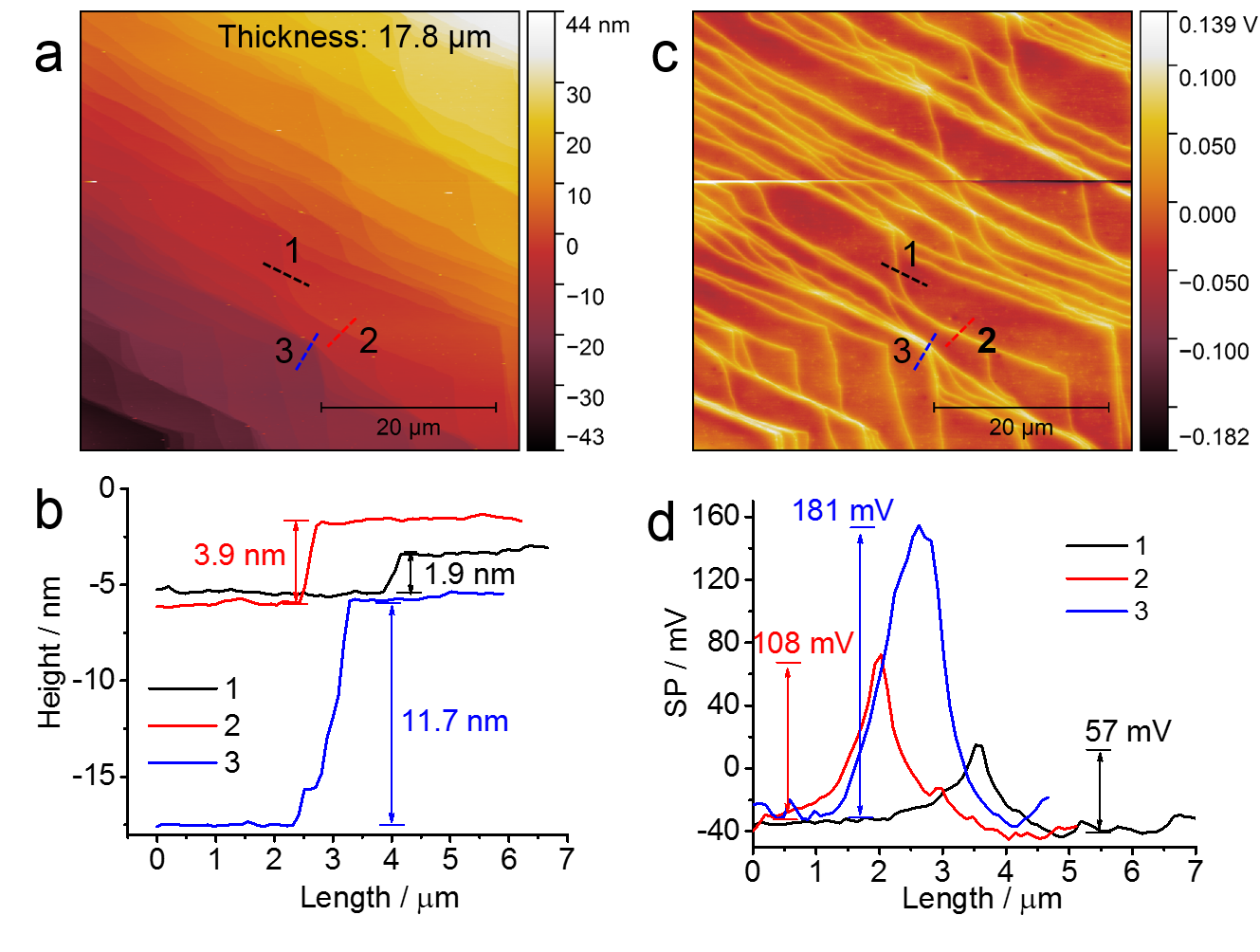


**Supplementary Figure 5 | Step edge potential increases for overlapping steps** **in a thick crystal. a**, AFM height image of a crystal with a thickness of 17.8 µm. **b**, Step height profiles along three dashed lines, corresponding to heights of 1.9 nm (black), 3.9 nm (red) and 11.7 nm (blue), respectively. The Cl_2_-NDI molecules stand upright on the crystal surface (*ab* face) with molecular length of ~1.9 nm. It can be deduced that the three dashed lines represent step edges of a monolayer (1), a double layers (2), and six molecular layers (3) overlapping, respectively. **c**, SKPM surface potential image. **d**, Corresponding surface potential profiles along the three dashed lines in **c**. The values of the surface potential increase where steps overlap. For a step edge monolayer, a positive potential of 57 mV was measured, which is very close to the average value of 60 mV. For a double-layer step edge, the surface potential increases to 108 mV. For a step edge of six overlapping layers, the surface potential profile exhibits a broad peak with a height of 181 mV.


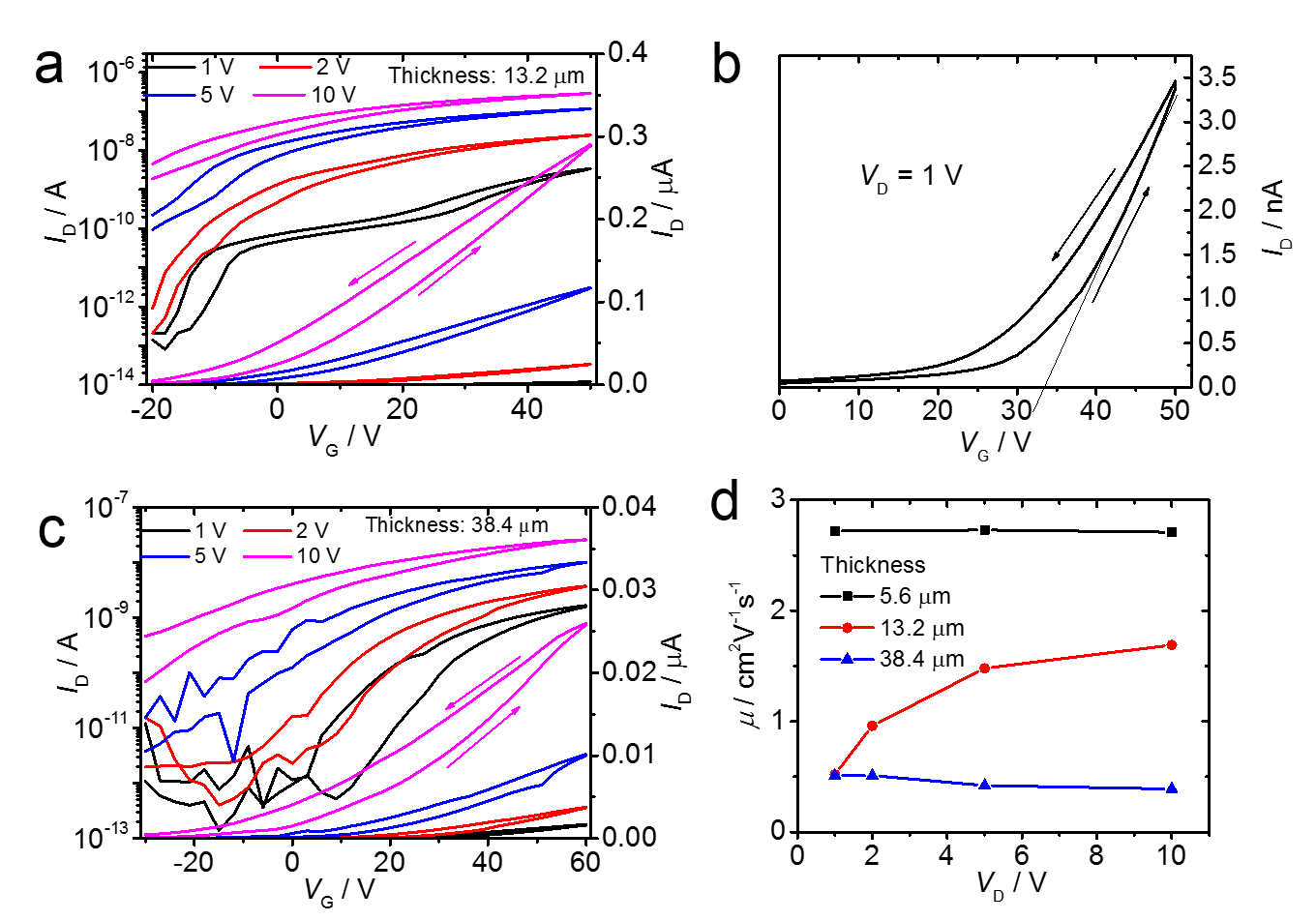


**Supplementary Figure 6 | Room temperature FET characteristics for Cl_2_-NDI crystals with intermediate (13.2 μm) and large (38.4 μm) thicknesses.** (**a**) Transfer curves at various *V*_D_ for a single crystal FET with a crystal thickness of 13.2 µm. (**b**) Corresponding magnified transfer curve at *V*_D_ = 1 V. (**c**) Transfer curves at various *V*_D_ for a thicker single crystal FET with a crystal thickness of 38.4 µm. Transfer curves of a thin single crystal (5.6 μm) were shown in Figure 1b. The channel length is fixed of 150 µm, the widths are 290 µm for the device in **a** and 100 µm for the device in **c**. (**d**) Corresponding mobility as a function of *V*_D_ for single crystal FETs with different thicknesses. For ideal thin single crystals (**d**, black squares), almost equal mobility is obtained at *V*_D_ of 1 V, 5 V and 10 V, indicating the influence of traps on charge transport is negligable. However, for the thick single crystal FETs (**d**, red circles), mobility increases with the increasing *V*_D_, showing a pronounced drain field effect. This indicates a portion of the mobile electrons are trapped and a large drain field effectively reduces the depth of traps and releases more trapped electrons. As the trap density further increases in the thicker crystal (**d**, blue triangles), a greater proportion of electrons will be captured traps, leading to a mobility of less than 1 cm^2^V^-1^s^-1^.


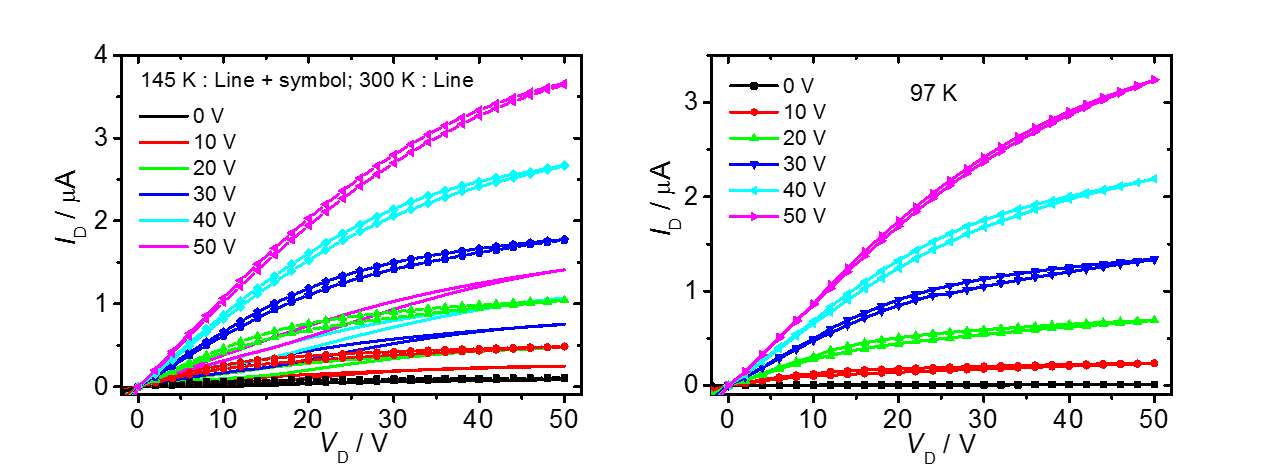
 **Supplementary Figure 7 | Output curves (*I*_D_-*V*_D_) corresponding to the device in Figure 3a at a temperature of 300 K, 145 K and 97 K, respectively.** Note: output curves at 97 K and 145 K were obtained in the process of warming up.


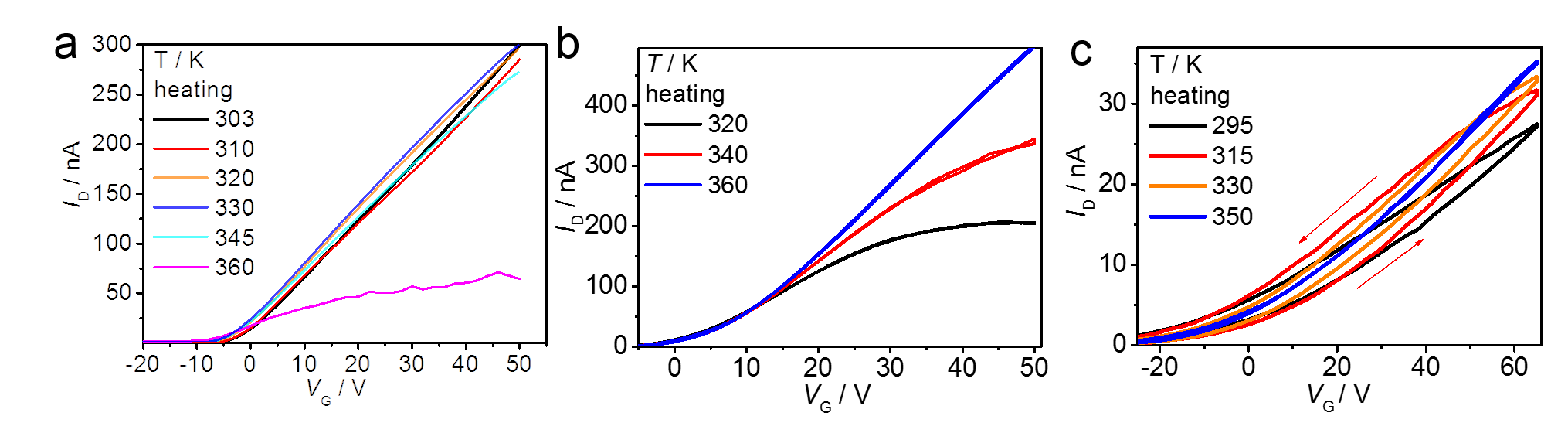


**Supplementary Figure 8 | Temperature dependent *I*_D_ -*V*_G_ characteristics above 300 K for the three crystals in Figures 3a, 3b, and 3c, respectively. a**, thin crystal: upon warming, mobility exhibits a slight decrease until the device broke at 360 K due to thermal expansion of the PDMS substrate. **b**, intermediate crystal: a significant mobility increase of about 1.6 times is observed from 1.67 cm^2^V^-1^s^-1^ at 300 K to 2.59 cm^2^V^-1^s^-1^ at 360 K. **c**, thick crystal: mobility increase by a factor of 1.32 from 0.39 cm^2^V^-1^s^-1^ at 300 K to 0.45 cm^2^V^-1^s^-1^ at 330 K.

**
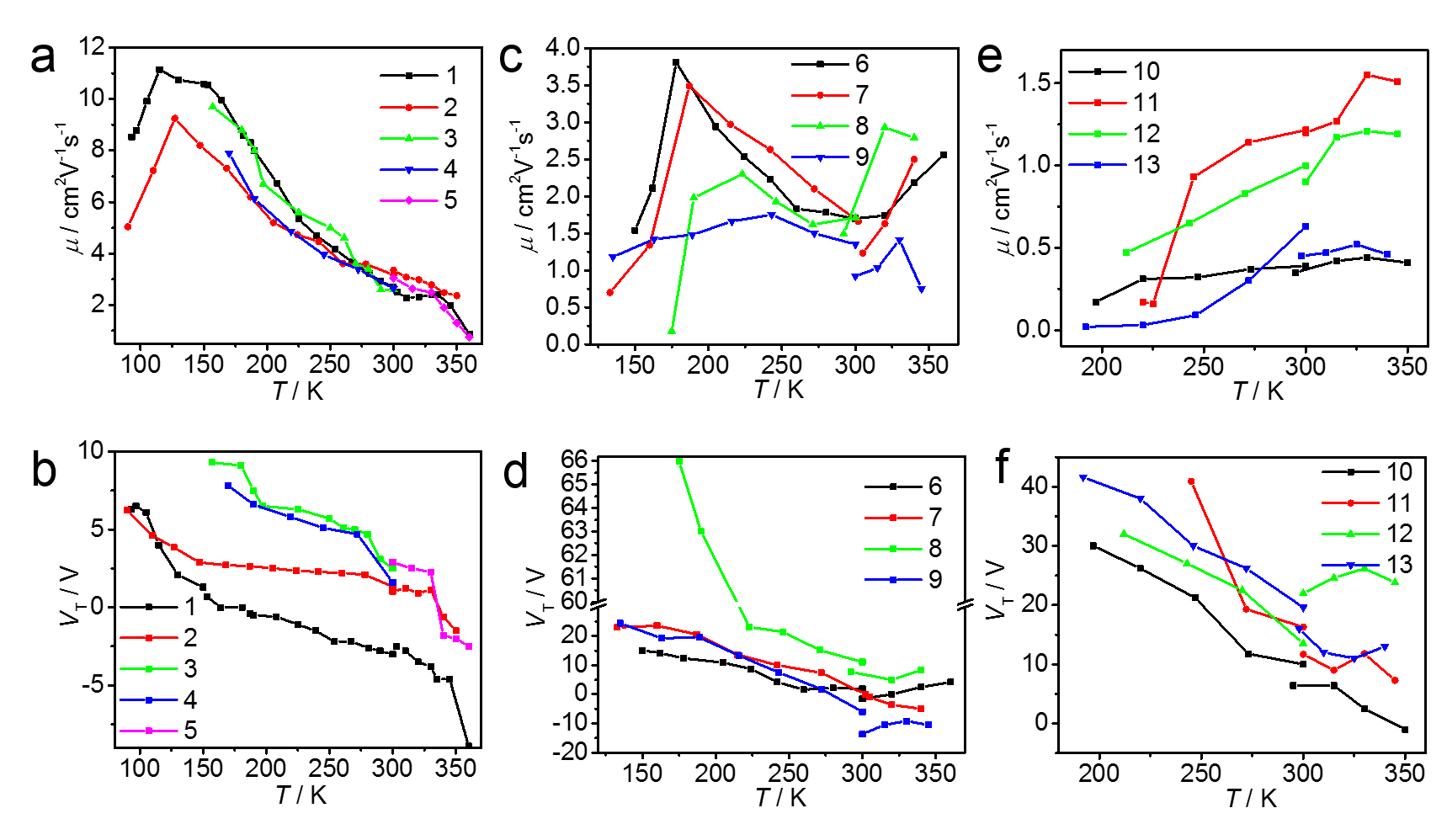
**

**Supplementary Figure 9 | Temperature dependent, four terminal mobility and threshold voltage measurements for Cl_2_-NDI single crystal FETs with different crystal thicknesses. a**, **b**, Mobility (*μ*) and threshold voltage (*V*_T_) versus temperature for single crystal FETs with a crystal thickness less than 6 µm. The devices exhibit *μ* > 2.6 cm^2^V^-1^s^-1^ at room temperature as well as band-like charge transport, i.e., mobility increases as temperature decreases. **c**, **d**, *μ* and *V*_T_ versus temperature for single crystal FETs with crystal thicknesses between 10 µm and 20 µm. Mobility is 1-2 cm^2^V^-1^s^-1^ at 300 K, and band-like charge transport is evident for *T* < 300 K, whereas activated transport holds for *T* > 300 K. **e**, **f**, Single crystal FETs with crystal thicknesses of more than 22 µm exhibit mobility ≤ 1.2 cm^2^V^-1^s^-1^ at 300 K and activated charge transport. In order to avoid thermal cycle induced extra cracks between adjacent steps and make sure thicker single crystal FETs are reproducible, devices with numbers of 10-13 were only cooled down to approximately 200 K.

**Supplementary Table 1 | Cl_2_-NDI FET performance and corresponding crystal thickness, µ4p-T relation (n), deep trap density (N_tr_) as well as thermal activation energy (E_A_).**

| Devices | µ ^a^ /  cm^2^V^-1^s^-1^ | µ ^b^ /  cm^2^V^-1^s^-1^ | Thickness /  µm | n ^c^ | N_tr_ ^d^/  10^11^cm^-2^eV^-1^ | E_A_ ^e^/  meV |
| --- | --- | --- | --- | --- | --- | --- |
| 1 | 2.71 | 11.14 (115K) | 5.6 | 2.05 (115-320K) | 4.8 | - |
| 2 | 3.11 | 9.25 (127K) | 4.1 | 1.52 (127-330K) | 2.3 | - |
| 3 | 2.68 | 9.70 (157K) | 4.8 | 2.03 (150-300K) | 5.7 | - |
| 4 | 2.64 | 7.89 (170K) | 3.1 | 1.85 (170-300K) | 5.1 | - |
| 5 | 3.05 | - | 3.7 | 2.10 (300-330K) | 2.7 | - |
| 6 | 1.67 | 3.81 (178K) | 13.2 | 1.63 (178-300K) | 13.1 | 61.8 (320-360K)  74.2 (150-178K) |
| 7 | 1.66 | 3.49 (187 K) | 14.6 | 1.52 (187-302K) | 18.6 | 63.0 (133-187 K) |
| 8 | 1.06 | 2.30 (223K) | 19.7 | 1.79 (223-271K) | 24.2 |  |
| 9 | 1.35 | 1.66 (216K) | 18.3 | 1.23 (243-300K) | 23.7 |  |
| 10 | 0.39 | - | 38.4 | - | 31.7 | 48.0 (197-330K) |
| 11 | 1.21 | - | 22.6 | - | 42.9 | 44.4 (245-345K) |
| 12 | 0.99 | - | 28.1 | - | 26.8 | 71.6 (212-315K) |
| 13 | 0.63 | - | 31.9 | - | 28.8 |  |

**a**, Four terminal mobility at room temperature. **b**, The highest mobility in the LT regime (T < ~300 K) and corresponding temperature. **c**, The values of n were extracted from the equation of $\mu\propto T^{-n}$ and corresponding temperature range of band-like charge transport. **d**, Deep trap density was extracted in the band-like region from ${{\partial N}_{\mathrm{tr}}}/{\partial E=(C_{i}/k_{B}e)\times(\partial V_{\mathrm{th}}/\partial T)}$. **e**, Thermal activation energy was extracted in the hopping region from the equation of $\mu\propto$ $\exp(-E_{A}/k_{B}T)$.


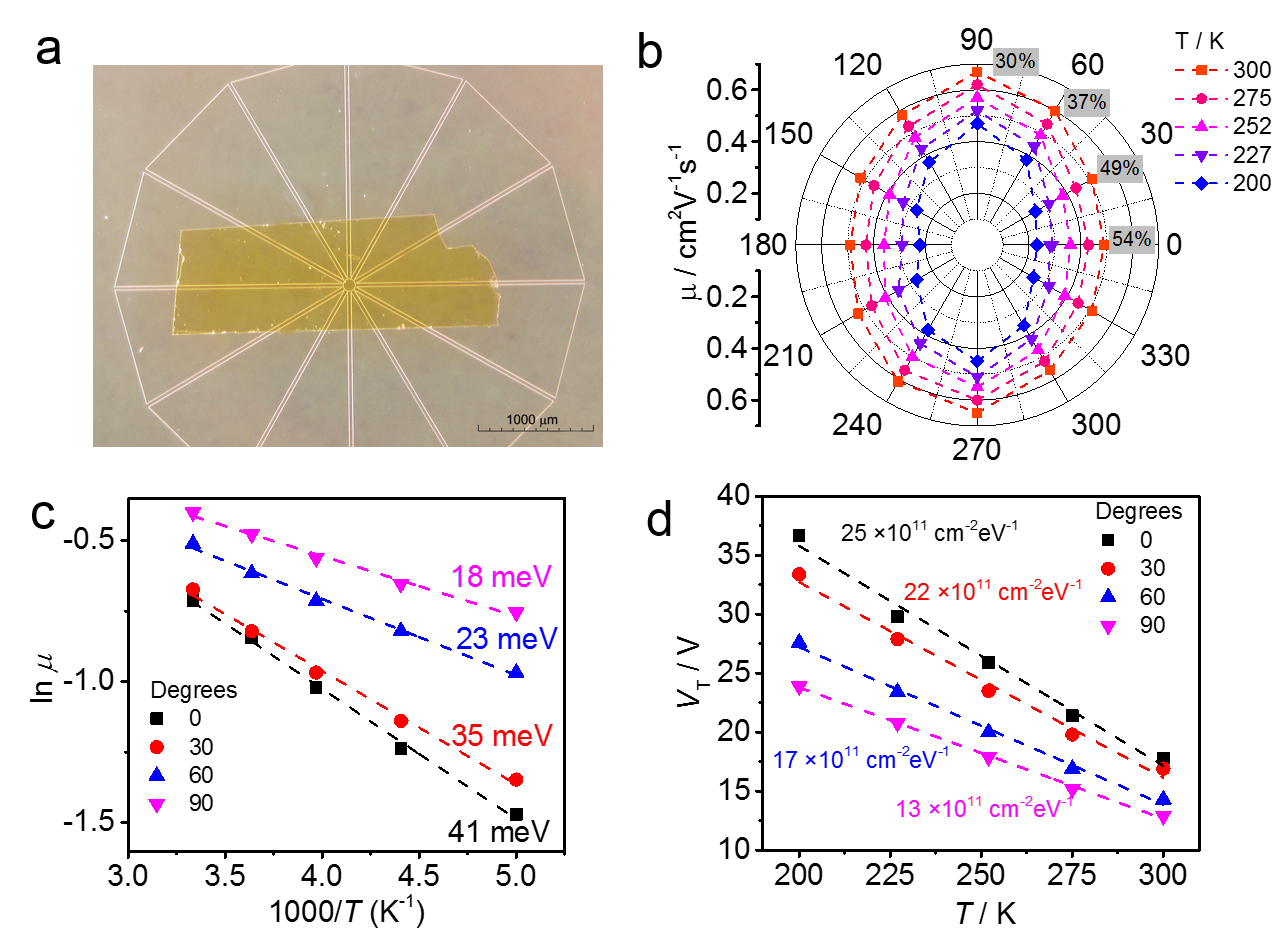


**Supplementary Figure 10 | The angular dependent device performance at different temperatures for a thicker (34.6 μm) Cl­_2_-NDI single crystal.** (**a**) Optical micrograph of Cl_2_-NDI on the fan-shaped contact pattern. (**b**) Corresponding anisotropic mobility distribution at different temperatures. Inserted percentages represent mobility degradation (∆*μ*/*μ*_300K_) in the respective directions. With cooling, the greatest mobility degradation was along 0° and the lowest along 90°, which is in agreement with oriented step edges shown in Figure 2b. (**c**, **d**) Step orientation dependent *ln* *μ* and *V*_T_ as a function of temperature. Inserted values represent activation energy and trap density that are extracted using $\mu\propto\exp(-E_{A}/k_{B}T)$ and ${{\partial N}_{tr}}/{\partial E=(C_{i}/k_{B}e)\times(\partial V_{th}/\partial T)}$, respectively. The highest activation energy of 41 meV and trap density of 25 ×10^11^ cm^-2^eV^-1^ are along the 0° direction.

**Supplementary Discussion 1**

**A model for the effect of terraces on Cl_2_-NDI** **single crystal FETs**

**1) Device model electrostatics and data analysis**

The fact that the threshold voltage for the thin-crystal Cl_2_-NDI OFETs is near zero or even slightly negative and that their conductance increases with increasing positive gate bias indicates that the thin Cl_2_-NDI crystals are conducting in equilibrium, and that the material is at least weakly *n*-type and not fully depleted. Allowing for some surface depletion at the top and bottom surfaces of the crystal slabs, the equilibrium sheet electron concentration, *n_s_*_0_, is given by,

$n_{s0}=n_{0}(d-d_{dt0}-d_{db0})$ (1)

Here *n*_0_ is the equilibrium bulk electron concentration, *d*, *d_dt_*_0_, and *d_db_*_0_ are the crystal and the top and bottom equilibrium depletion layer thicknesses, respectively. Assuming that the top and bottom surfaces are similar, we expect $d_{dt0}\cong d_{db0}$_._

The built-in potential associated with the depletion layer at the bottom surface (facing the gate) may be written as,

$U_{bi0}=\frac{2\pi e^{2}}{\kappa_{0}}n_{0}{d_{db0}}^{2}=\frac{\pi e^{2}}{2\kappa_{0}}n_{0}d^{2}{(1-\frac{n_{s0}}{n_{0}d})}^{2}$ (2)

where *e* is the elementary charge, *κ*_0_ is the dielectric constant of the material and we used the assumption of similar surfaces in the last step.

The equilibrium Fermi level in the bulk lies below the LUMO level by an energy $\Delta\epsilon_{F}=kTln(\frac{N_{0}}{n_{0}})$. Here *kT* the thermal energy, and the effective density of states, *N*_0_, is taken to be twice (to account for spin) the molecular density. Therefore, knowing the work function of the contact metal (Au, *W* = 5.1eV) and the electron affinity of Cl_2_-NDI (Χ = 4eV) yields the following relationship between *n_s_*_0_ and *n*_0_:

$\ln\left( \frac{N_{0}}{n_{0}} \right)=\frac{W-X}{kT}-\frac{U_{bi0}}{kT}$ (3)

We assume here that there is no built-in electric field across the air gap, i.e. that the Cl_2_-NDI crystal is charge neutral in equilibrium. Extracting *n_s_*_o_ from measured OFET data for a thin crystal (e.g. *d* = 5.6 μm) for vanishing gate bias, and taking the top and bottom depletion layers to be equal, we arrive at *n*_0_ = 3.5x10^13^ cm^-3^. We shall assume that the equilibrium bulk electron concentration is the same for all crystals.

Continuing with this simple, one-dimensional device model, we examine the surface more closely. We shall assume that relatively deep surface traps are present and that their population by electrons controls the depletion. The sheet density of occupied traps is *N*_t_, and we shall assume that all traps are populated by electrons, except perhaps traps that are in such close proximity that the electrons’ mutual Coulomb repulsion suppresses their simultaneous occupation. We also allow for a bias voltage to the gate, *V*_G_, assuming that the crystal is grounded. Retaining the assumption of local equilibrium in the Cl_2_-NDI (and in the gate contact), we generalize eq. (3) to the following relationship:

$\ln\left( \frac{N_{0}}{n_{0}} \right)=\frac{W-X}{kT}-\frac{U_{\mathrm{bi}}}{kT}-\frac{eV_{G}}{kT}-\frac{\Delta U}{kT}$ (4)

Here we express the electrostatic potential drop between the crystal surface and the gate by,

$\Delta U=\frac{e^{2}}{C}[n_{0}\left( d-d_{\mathrm{dt}} \right)-n_{s}-N_{t}]$ (5)

where *C* is the capacitance per unit area between the gate and the Cl_2_-NDI crystal.


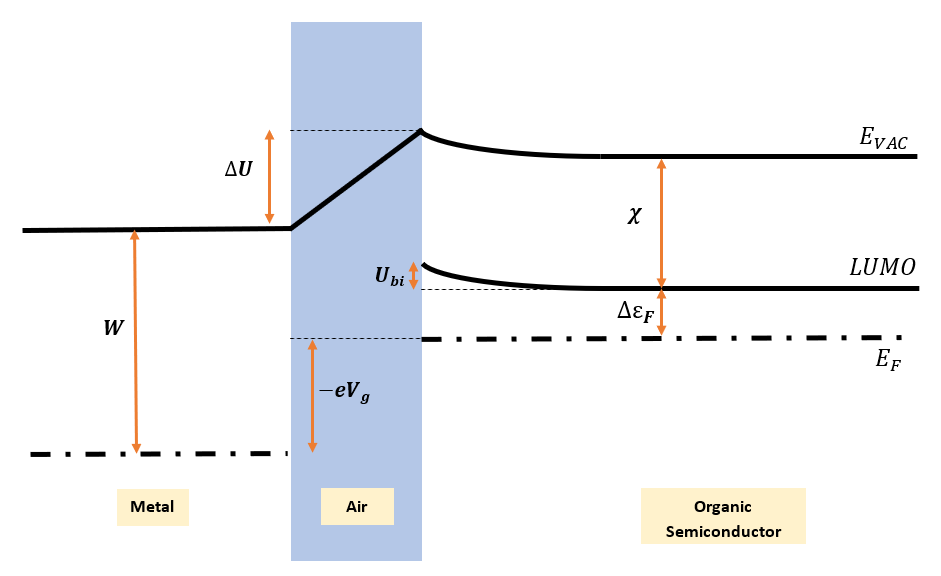


**Supplementary Figure 11 | Schematic band diagram of the metal/air/** **Cl_2_-NDI** **structure.**

The thick Cl_2_-NDI crystals are fully depleted in equilibrium and OFETs made from them therefore have a positive threshold voltage. Hence, the arguments made above do not apply for equilibrium. However, for large positive gate voltages (well above threshold, i.e. when the crystal is only partially depleted) the model represented by Supplementary Figure 11 still applies, and we expect the last two terms in eq. (4) to be to much larger than all the other terms. Therefore, $\Delta U \approx-eV_{G}$ and we conclude that,

$n_{s}\approx n_{0}\left( d-d_{\mathrm{dt}} \right)-N_{t}+(\frac{C}{e})V_{G}$ (6)

Analogous to the arguments made above for equilibrium, if top and bottom crystal surfaces have the same densities of traps we may write: $n_{0}d_{\mathrm{dt}}=N_{t}$.

The sheet carrier concentration is readily determined from the measured drain current at a given gate voltage and small drain voltage.

Comparing two devices of different thickness, but with the same applied gate voltages, we find from eq. (6) that the difference in their sheet electron concentrations is given by,

$n_{s2}-n_{s1}=n_{0}\left( d_{2}-d_{1} \right)-2N_{t2}+2N_{t1}$ (7)

We associate the surface traps on the Cl_2_-NDI crystals with steps between terraces. We take the steps to be aligned parallel to each other. Hence, we can relate the surface density of traps to the lineal densities of traps along a step and the lineal density of steps across the surface. Both of these quantities are simply expressed in terms of the mean distances between traps along a step and between steps,

$N_{t}=\frac{1}{\Delta x\Delta y}$ (8)

Here we define the *x*-direction as perpendicular to the steps and the *y*-direction as parallel to the steps. AFM measurements yield $1/\Delta x$, which was found to be approximately proportional to the crystal thickness, $\frac{1}{\Delta x}=\alpha d$. We therefore conclude that,

$n_{s2}-n_{s1}=(n_{0}-2\frac{\alpha}{\Delta y})(d_{2}-d_{1})$ (9)

Taking as a specific example the crystals of thickness 5.6μm and 24μm with applied gate bias of 40V and the experimentally determined parameter *α* = 3.7x10^6^ cm^-2^ allows us to determine the mean distance between filled traps along a step as Δ*y* = 1.3x10^-7^ cm. That value is not much larger than the lattice constants of NDI, i.e. there is a filled trap associated with nearly every molecule along a step. We view this result as an indication that the traps are probably an intrinsic property of the steps, rather than an effect associated with surface contamination or aggregation of foreign molecules at the steps.

We may also check the extrapolated variation of the threshold voltage (*n*_s_ = 0) and we find:

$\frac{\partial V_{T}}{\partial d}\approx-\left( \frac{e}{C} \right)\frac{n_{s2}-n_{s1}}{d_{2}-d_{1}}=1.9x{10}^{4}V {cm}^{-1}$. This value is in close agreement with the experimental result for small drain bias and *d* between 2 μm and 20 μm.

Since the traps are assumed to be located along the steps the resulting surface charge density is not uniform. In all cases we have $\Delta x\gg\Delta y$. The one-dimensional model used above represents only an average over *x*, while the true depletion region displays the distribution of steps across the surface and varies with *x* and *z* (perpendicular to the surface) on length scales given by Δ*x* and *d*_d_, which are both in the range of μm.

For thin crystals under very large positive gate bias, mobile electrons accumulate at the bottom surface (facing the gate) and the distribution of filled traps will merely modulate the accumulated mobile electron density, rather than give rise to true depletion regions. The length scale of the modulation is then given by an effective Debye length, which in the cases of strongest accumulation examined here is estimated to be on the order of 0.1 μm. In all of these cases, however, electrostatic potentials associated with the distribution of filled traps are expected to impact the electron transport - relatively weakly if transport is parallel to the steps (*y*) and strongly if it is perpendicular to the steps (*x*).

**2) Model for the formation of traps at surface terrace steps**

In the following we propose a simple conceptual model for the origin of traps at steps between surface terraces. Let’s first consider a planar surface of a perfect semiconductor. Inevitably, such a surface gives rise to a microscopic dipole layer because the negative charge density of the electron distribution extends beyond the positive charge density of the ion cores into the vacuum. In a simple model, it is this dipole layer that determines the alignment of the electronic structure of the material with the vacuum level above the surface. Choosing *z* as the axis perpendicular to the surface, the dipole layer may be characterized by a dipole density *P_z_*, which is uniform on the surface.

A perturbation of the planarity of the surface, such as a step between two adjacent planar terraces implies a locally different dipole layer, *P_z,step_*. If the step is located at *x* = 0 and runs parallel to the *y*-axis, this different dipole density will be restricted to the vicinity of *x* = 0 and it will be translational invariant along *y*. We may define the variation in the dipole density on the surface of the crystal as *ΔP_z_* = *P_z,step_* – P_z_. Since we are interested in the spatial variation of the surface potential and not in its absolute value, we will consider only *ΔP_z_* in the following discussion. *ΔP_z_* consists of a row of dipoles on the face of each step between adjacent terraces. Averaging along the *y*-direction, it can be modeled as two parallel line charges of lineal density *ρ* and opposite sign that are separated in the *z*-direction by 2*δ*. The electron potential energy of such a row of dipoles is easily calculated.

$U\left( x,z \right)=(\frac{e\rho}{\kappa_{\mathrm{eff}}})ln(\frac{x^{2}+\left( z-\delta\right)^{2}}{x^{2}+\left( z+\delta\right)^{2}})$ (10)

It is plotted as a function of *x* for different values of *z* in Supplementary Figure 12, assuming that the dipoles are oriented up, i.e. out of the semiconductor. The potential is negative (attractive for electrons) above the surface (*z* > *δ*), but positive below, i.e. in the region of the material where a conducting channel forms. The potential falls off rather slowly (logarithmically), but on a microscopic length scale (given by *δ*) with increasing *x* and *z*. The strength of the potential is given by the lineal charge density associated with the dipoles. Taking that quantity as of order *e*/*a*, where *a* is a lattice constant, the potential is quite strong (*eρ*/*κ_eff_* ~ 1.3 eV) over microscopic distances (*δ* ~ 0.5 nm) and can easily lead to bound electron states immediately above the nominal surface. (The effective dielectric constant is taken as the arithmetic average of the dielectric constants of air and the crystal).





**Supplementary Figure 12 |** **Dipole potential in units of *eρ*/*κ_eff_*.** The coordinates *x* and *z* are in units of *δ*.

Viewing this bound state as a deep trap on the surface the repulsive part of the dipole potential immediately inside the material is of little consequence as that region will be depleted of electrons if the trap is populated. As argued above, this depletion (or the spatially modulated accumulation for very large gate bias) varies on a macroscopic length scale.

Allowing for averaging of the dipole and depletion region potentials along the *y*-direction $(\Delta y\ll\Delta x)$, the resulting total potential does not depend on *y*. Therefore, no force on the electrons is exerted in that direction and their dynamics involving motion along *y* is unaffected. However, electron motion along the *x*-direction is strongly affected by the *x*-dependent potential that forms barriers for the electrons. If the barriers are relatively low and the transport is ‘band-like’ they can be thought of as scattering agents. However, if they are large, the transport will become activated, even if that is not the case for the perfectly flat surface.


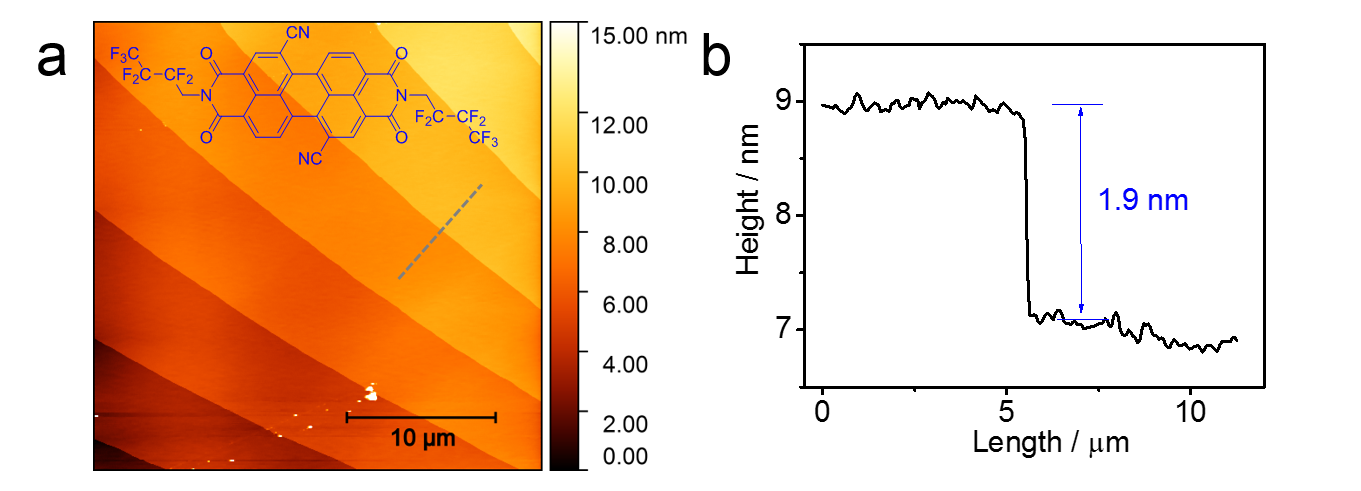


**Supplementary Figure 13 | Corresponding AFM height profiles of a PDIF-CN_2_ single crystal.** AFM height image (**a**) and step height (**b**) profile along the gray dashed line. The step height is ~ 1.9 nm, which matches well with *c*-axis of crystal parameter^2,3^.

**Supplementary Figure 14 |** **Mobility distribution and step density at the surface of PDIF-CN_2_ crystals as a function of crystal thickness.** The mobility was extracted from 35 devices at *V*_D_ = 10 V, and a step density was measured at the surface of 25 crystals by AFM. The number of steps was counted along the direction of the crystal’s long-axis. Step density is proportional to crystal thickness. Compared to Cl_2-_NDI, PDIF-CN_2_ transistors show a steeper mobility degradation as crystal thickness increases, because of a much higher surface potential, ~ 100 mV, at the step edge.


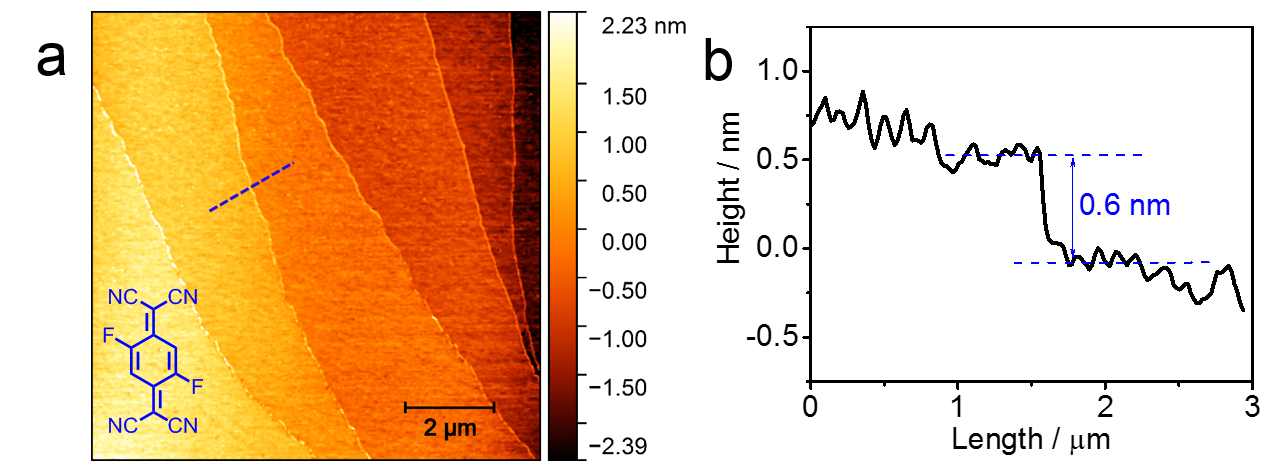


**Supplementary Figure 15 | Step edges on the surface of a F_2_-TCNQ single crystal.** AFM height image (**a**) and step height (**b**) profile along the blue dashed line. The step height is ~ 0.6 nm^4^.


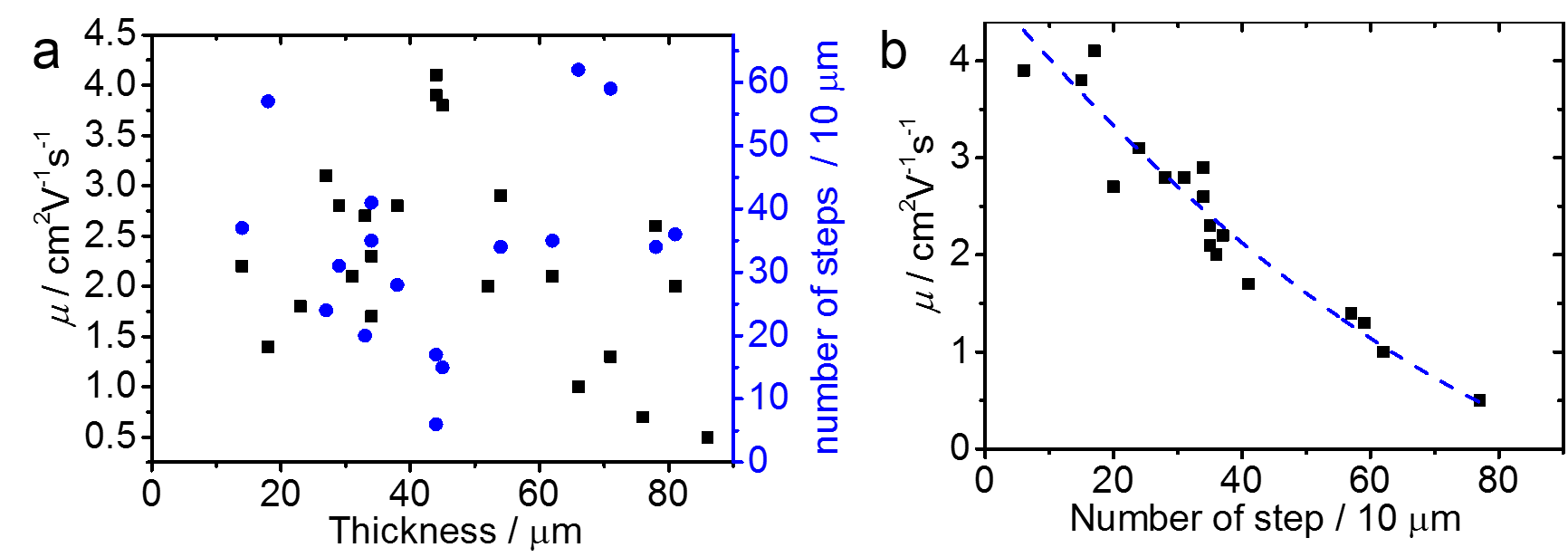


**Supplementary Figure 16 |** **Mobility and step density distribution for F_2_-TCNQ crystals with different thicknesses.** The mobility was extracted from 22 crystals at *V*_D_ = 10 V, and step density was measured at the surface of 18 crystals by AFM. The number of steps was counted along the direction of the crystal’s long-axis. Different from Cl_2_-NDI and PDIF-CN_2_ crystals, F_2_-TCNQ crystals exhibit crystal thickness independent step density. Even so, the trend of mobility decrease with increasing step density can still be observed.


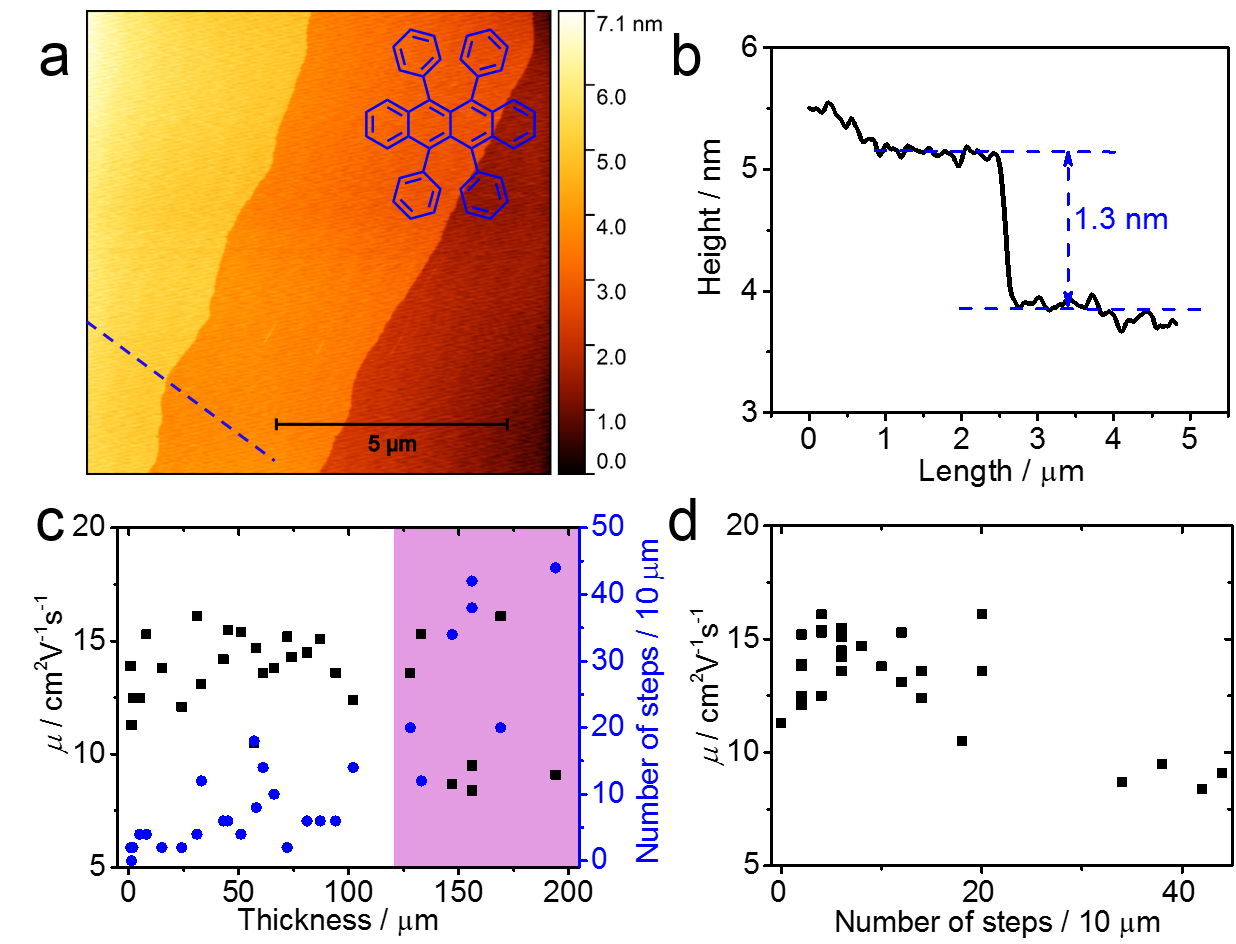


**Supplementary Figure 17 | Step edges on rubrene single crystal surfaces and corresponding FET mobility.** AFM height image (**a**) and corresponding height profile along the blue dashed line (**b**). The step edge height is 1.3 nm, corresponding to one molecular layer^5^. **c**, Mobility and step density distribution as a function of crystal thickness. The mobility was extracted from 29 crystals at *V*_D_ = 1 V. **d**, Mobility versus step density. Mobility dependence on step density is weak because the step edge potential is very small (~ -10 mV), see Figure 5c.


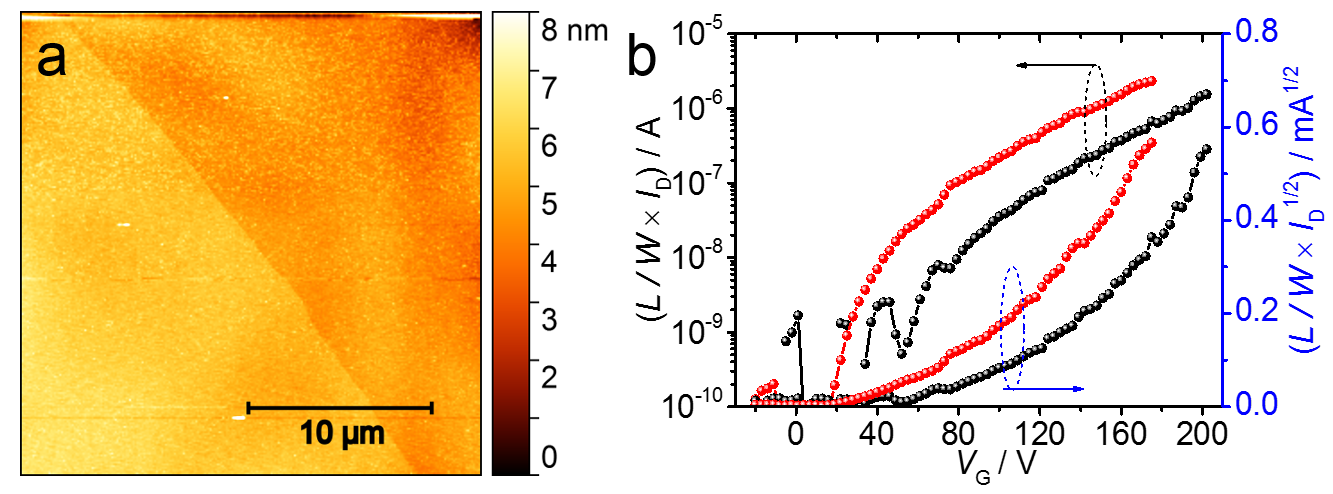


**Supplementary Figure 18 | Step edge on C_60_ single crystal and corresponding FET measurements. a**, AFM image of a C_60_ single crystal with low step density. **b**, Transfer curves of C_60_ single crystal devices. The red curve corresponds to the crystal with high step density in Figure 5f and peak electron mobility is 2.6 cm^2^V^-1^s^-1^ at *V*_D_ and *V*_G_ of 175 V. Black curve corresponds to the crystal with low step density in (**a**) and peak mobility is 2.0 cm^2^V^-1^s^-1^ at *V*_D_ and *V*_G_ of 200 V. The FET performance does not seem strongly dependent on step density.

**Supplementary Discussion 2**

**Microelectrostatic modeling of step edges in molecular crystals**

As discussed in the main text, several effects can contribute to the surface potential signature of step edges, including disorder in molecular orientations and strain. Here we focus on an intrinsic mechanism that rationalizes surface potential variations in terms of uncompensated dipoles at idealized (defect- and strain-free) step edges.

**Supplementary Table 2 | Quadrupole tensor of PDI and NDI derivatives.** Principal components of the quadrupole tensor in D·Å units.

|  | Q_1_ | Q_2_ | Q_3_ |
| --- | --- | --- | --- |
| Cl_2_-NDI | -26.1 | -0.7 | 26.8 |
| C_6_-NDI | -16.2 | -2.7 | 18.9 |
| PDIF-CN_2_ | -38.8 | -10.8 | 49.5 |


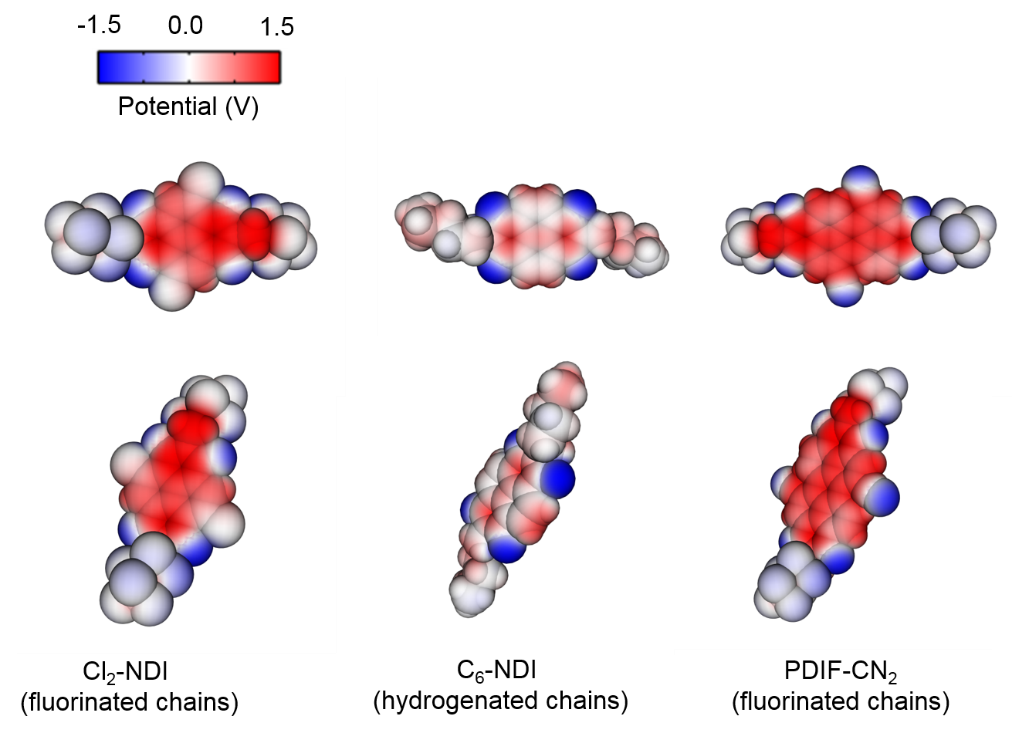


**Supplementary Figure 19 | Molecular electrostatic potential.** Electrostatic potential color-coded on the molecular van der Waals surface. The potential is calculated with ESP atomic charges obtained from density functional theory calculations at the PBE0/6-311++G(p,d) level of theory.

In their respective crystal structures, Cl_2_-NDI, C_6_-NDI and PDIF-CN_2_ molecules lie at inversion centers, hence featuring a vanishing dipole moment. Their leading electrical multipole is therefore the quadrupole *Q*, whose components for the three molecules are listed in Table S2. The molecular electrostatic potential, shown in Supplementary Figure 19, and consequently the quadrupole, strongly depend on the chemical groups functionalizing the naphthalene or perylene core. For the three molecules we consider, oxygen atoms of diimide groups appear as negative-potential “blue” spots owing to their electron attracting character. Fluorinated side chains and electron-withdrawing bay substituents (Cl, CN) further deplete the electron density of the π-conjugated region, leading to strikingly different potential on molecular cores of Cl_2_-NDI and PDIF-CN_2_ with respect to C_6_-NDI.


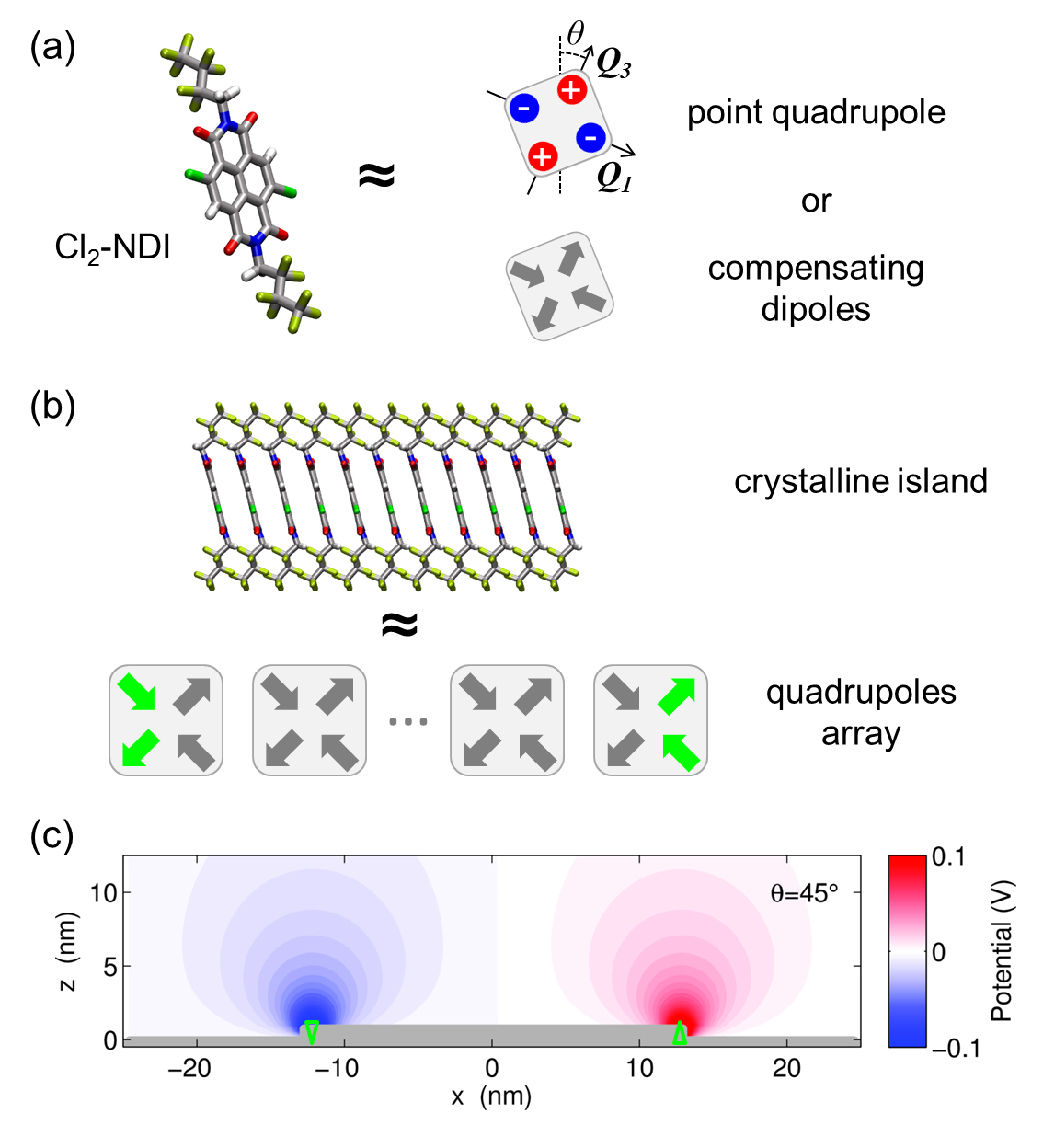


**Supplementary Figure 20 | Molecular quadrupole model for step edge potential. a** The charge density of centrosymmetric molecules (Cl_2_-NDI in the specific example) is approximated as a point quadrupole that can be also represented as a set of compensating dipoles. The angle *θ* expresses the tilt of the quadrupole largest component with respect to the plane normal *z*. **b** A crystalline island is seen as an array of quadrupoles. Intramolecular dipoles compensate in the inner part of the islands (gray arrows) but they do not at its edges, leading to net dipole of opposite polarity at the two ends (green arrows). **c** Electrostatic potential calculated with the quadrupole model for *θ*=45 degrees. In this case the step-edge dipoles point vertical.


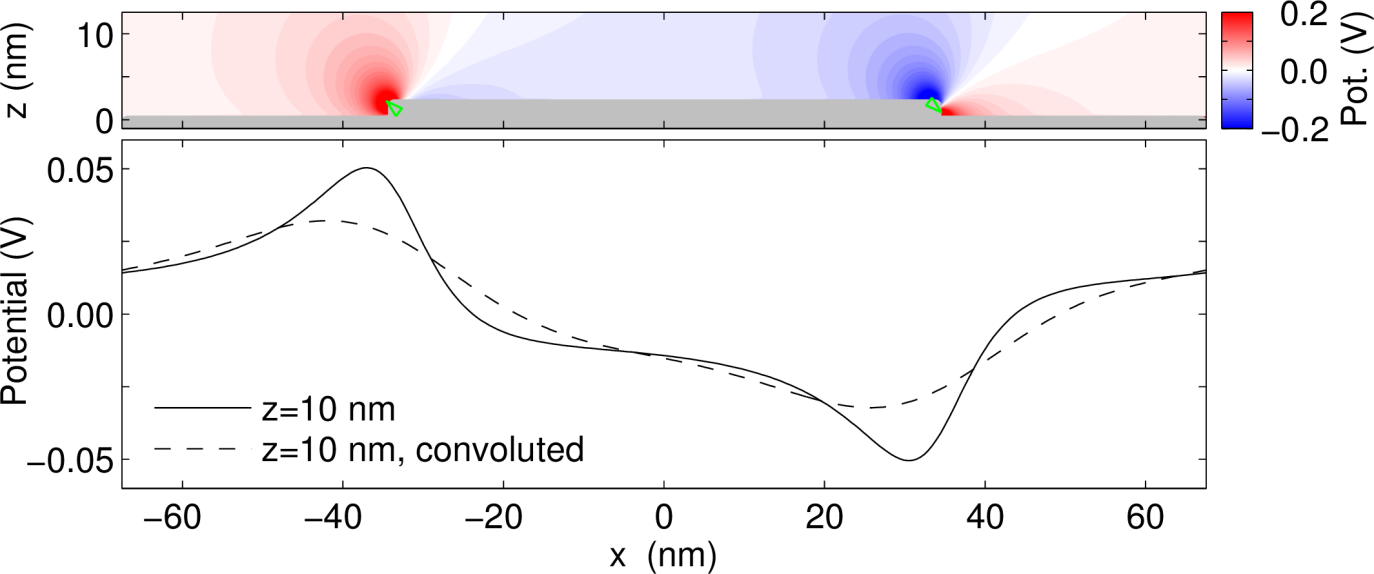


**Supplementary Figure 21 | Electrostatic potential at Cl­_2_-NDI step edges.** The upper panel shows the electrostatic potential in the *xz* plane generated by a crystalline island with right and left hand side step edges. The gray-shaded area shows the island and the underlying complete molecular layer. Effective dipoles at step edges are sketched as green triangles. The bottom panel shows potential profile at constant *z=10* nm (full line). The dashed line shows the potential convoluted with a Gaussian function (standard deviation 10 nm), mimicking the limited lateral resolution of the AFM tip. The *x* and *z* axis correspond to the [110] and [001] crystallographic directions, respectively.


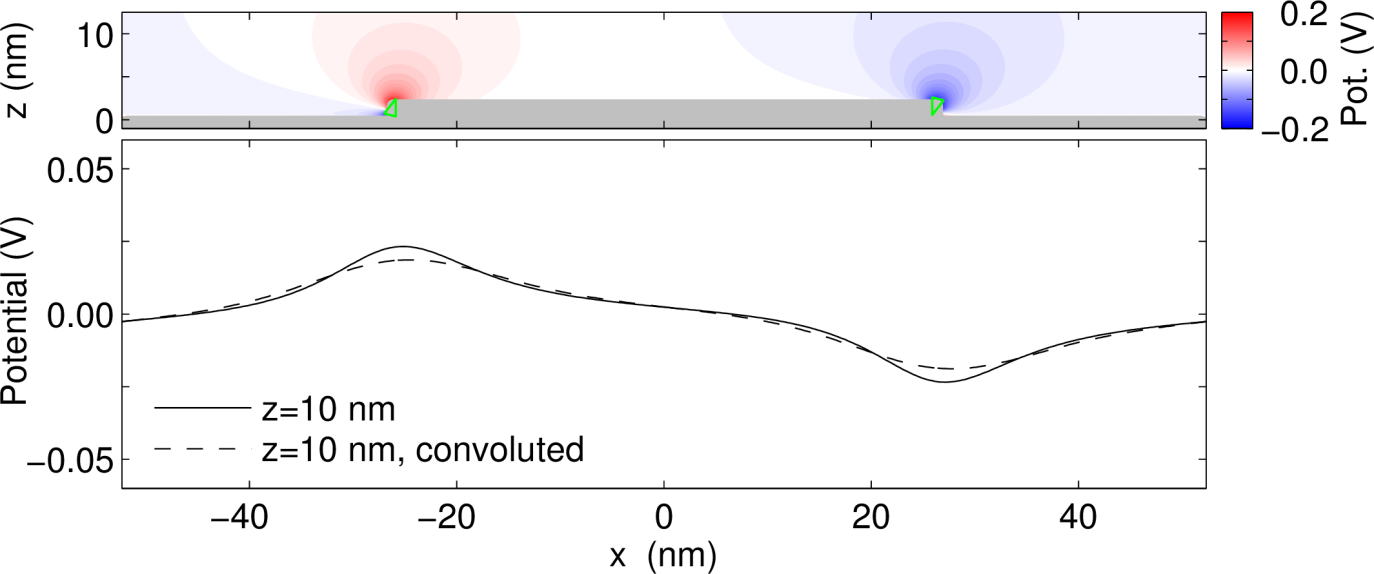


**Supplementary Figure 22 | Electrostatic potential at PDIF-CN_2_ step edges.** Same as Supplementary Figure 21 for PDIF-CN_2_. The potential due to nearly upright edge dipoles peaks at the step edges. The *x* and *z* axis correspond to the [100] and [001] crystallographic directions, respectively.


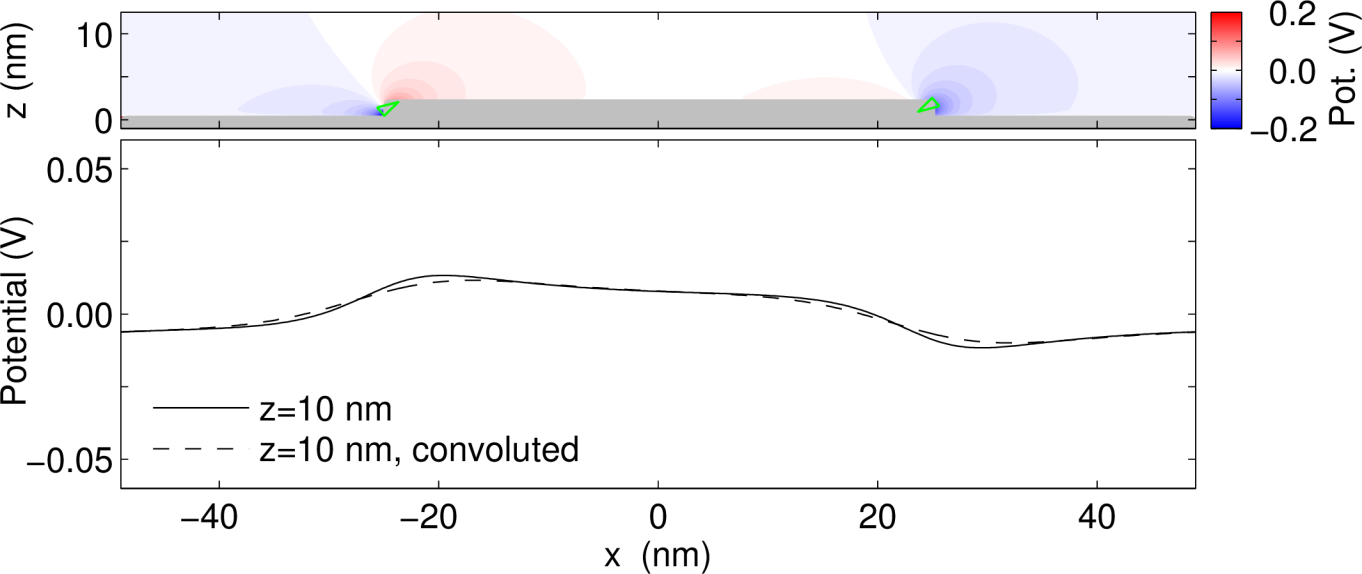


**Supplementary Figure 23 | Electrostatic potential at C_6_-NDI step edges.** Same as Supplementary Figure 21 for the C_6_-NDI. The electrostatic potential variations at step edges are smaller and smoother than in Cl_2_-NDI, owing to the smaller quadrupole moment (see Table S2) and of the almost lying step dipoles (green triangles). The *x* and *z* axis correspond to the [100] and [001] crystallographic directions, respectively.

The importance of electrostatic phenomena arising from molecular quadrupoles has been acknowledged only recently. It has been shown that charge-quadrupole interactions can impact charge transport levels in organic semiconductors by several tenths of an eV^6,7^, yet the electrostatic potential of complete molecular layers presents a rapid exponential decay with distance. As we will show below, the discontinuity introduced by step edges is responsible for long-ranged electrostatic potential arising from the lack of compensation of quadrupole moments.

Supplementary Figures 20-23 show the electrostatic potential calculated with an atomistic point charge model at step edges of Cl_2_-NDI, PDIF-CN_2_ and C_6_-NDI, respectively. Atomic charges from electrostatic potential fitting (ESP scheme)^8^ have been obtained from density functional theory calculations (PBE0/6-311++G(p,d) level of theory) performed with the Gaussian09 code^9^. We considered model morphologies built from the respective X-ray crystal structures with step edges in the (001) surfaces and step-normal direction along the [110], [100] and [100] crystallographic directions for Cl_2_-NDI^1^, C_6_-NDI^10^ and PDIF-CN_2_^2^, respectively. The potential has been calculated with Parry 2D-periodic electrostatic sums^11^ as implemented in the GULP package^12^, ensuring converged potential at arbitrary distance from the surface. Because of 2D periodic boundary conditions, our simulation setup describes an infinite 1D array of crystalline islands with periodicity much larger than the crystal lattice constant normal to the step. The system has infinite extension along the step direction.

The potential at the step edges of Cl_2_-NDI, shown in the upper panel of Supplementary Figure 21, is characterized by remarkable features of the same magnitude but of opposite sign at the two right and left step of the island that resemble the potential of two tilted dipoles pointing in opposite directions (green triangles). To better quantify the variations of the potential, the bottom panel shows a scan at 10 nm constant distance, corresponding to a typical tip-surface distance in a SKPM experiment. The potential at step edges of PDIF-CN_2_ in Supplementary Figure 22 is similar, although its magnitude is smaller and the two “effective dipoles” are more upright. For C_6_-NDI in Supplementary Figure 23 the electrostatic potential variations across step are below 20 meV and the end dipoles are lay almost parallel to the substrate.

The magnitude of the calculated potential at step edges compares favorably with SKPM measurements for the three systems. The potential scans at 10 nm distance from the surface present variations that are in semi-quantitative agreement with measurements for Cl_2_-NDI and PDIF-CN_2_, while the almost flat potential calculated for C_6_-NDI is consistent with the negligible SKPM signal. Structural reorganization, disorder and oxidation at step edges, observed in experiments but not considered in the model, are also expected to contribute to the potential, hindering a full quantitative comparison.

An important issue for the theory-experiment comparison concerns the sign of the potential. Calculations target a crystalline island prescribing opposite potential at the two steps, while the single crystals grown and measured in this work present terraced surfaces with always either ascending or descending steps and potential variations always of the same sign. In order to discriminate which step is more likely to occur in real samples, we performed quantum mechanical calculations to determine the adhesion energy of the left and right side of the island. Specifically, we performed semi-empirical Hartree-Fock calculations (PM6-D3H4 parametrization including dispersion corrections^13^, MOPAC package^14^) with 2D periodic boundary conditions. The adhesion energy $E_{ad}^{l/r}$ is defined as the energy gained upon adding a linear array of molecules at the left and right of a perfect crystalline island. We found that the adhesion energy is larger for the right than for the left step in both Cl_2_-NDI ($E_{ad}^{r}-E_{ad}^{l}$=2.8 meV/molecule) and PDIF-CN_2_ ($E_{ad}^{r}-E_{ad}^{l}$=19.2 meV/molecule). This suggests a faster kinetics for the growth of right edges and the preferential occurrence of left edges in terraced surfaces, although the small differences cannot rule out entropic effects at finite temperature. We hence conclude that left step edges with positive surface potential (see Supplementary Figures 20 and 21) dominate, in accordance with SKPM measurements (see Figure 5).

In order to understand the origin of the opposite dipole-like field at the two step edges and to disentangle the effect of the quadrupole magnitude versus molecular orientation, we resort to a simpler model where a fictitious molecule is represented by a point quadrupole. Specifically, we consider a traceless quadrupole with principal components *Q_3_=-Q_1_=10* D·Å (*Q_2_=0*) and build step edges on the (001) surface of an orthorhombic lattice (cell parameters: *a=b=5* Å*, c=10* Å) with one molecule per cell. The step is taken normal to the [100] crystallographic direction. The orientation of the fictitious molecule is varied by tuning on the angle θ formed by the principal direction ***Q_3_*** and the plane normal. Reciprocal space electrostatic sums have been evaluated as in the atomistic model by replacing quadrupoles with four point charges at small distance.

As illustrated in the panel a of Supplementary Figure 20, a given molecule is approximated by a point quadrupole with tilt angle *θ* or, equivalently, by a set of compensating dipoles. For our simplified quadrupole layout four coplanar dipoles are sufficient, while a larger number of compensating dipoles in the 3D space are required in the more general case, or to describe electrical multipoles higher than the quadrupole. A crystalline island can be therefore seen as an array of quadrupoles seen as compensating dipoles as sketched in panel b. The dipoles in the central part of the island (gray arrows) compensate each other, leading to an exponential decay of the potential with characteristic length commensurate to the in-plane periodicity. On the other hand, the dipoles at the two step edges (green arrows) remain uncompensated, resulting in a net effective dipoles pointing in opposite directions, explaining the results for atomistic systems in Supplementary Figures 20-23. Supplementary Figure 20c shows the potential obtained with the point quadrupole model for *θ*=45 degrees. In this case, the edge dipoles point exactly vertical (green triangles), upward or downward, exerting a potential with the characteristic angular dependence prescribed for a dipole.

Two observations must be made. First, since our model system is two dimensional and periodically repeated along the *xz* plane nomal, there is actually a linear array of dipoles propagating along the step edge. This leads to a potential scaling with distance as *r^-1^*_,_ instead that the *r^-2^* or *r^-3^* dependence expected for an isolated dipole or quadrupole. This explains the long range nature of the step edge potential and the SKPM signal dependence with the tip-surface distance (data now shown). The second observation concerns the local nature of the step edge potential. Indeed, while our simulations are bound to 2D periodic boundary conditions and cannot describe separately the left and right steps of an island, the effect of each edge is actually distinct and can be decoupled in the limit of infinite distance between the two steps. The local potential calculated at one edge therefore applies also to step edges in terraced crystal facets, such as those measured in real samples.


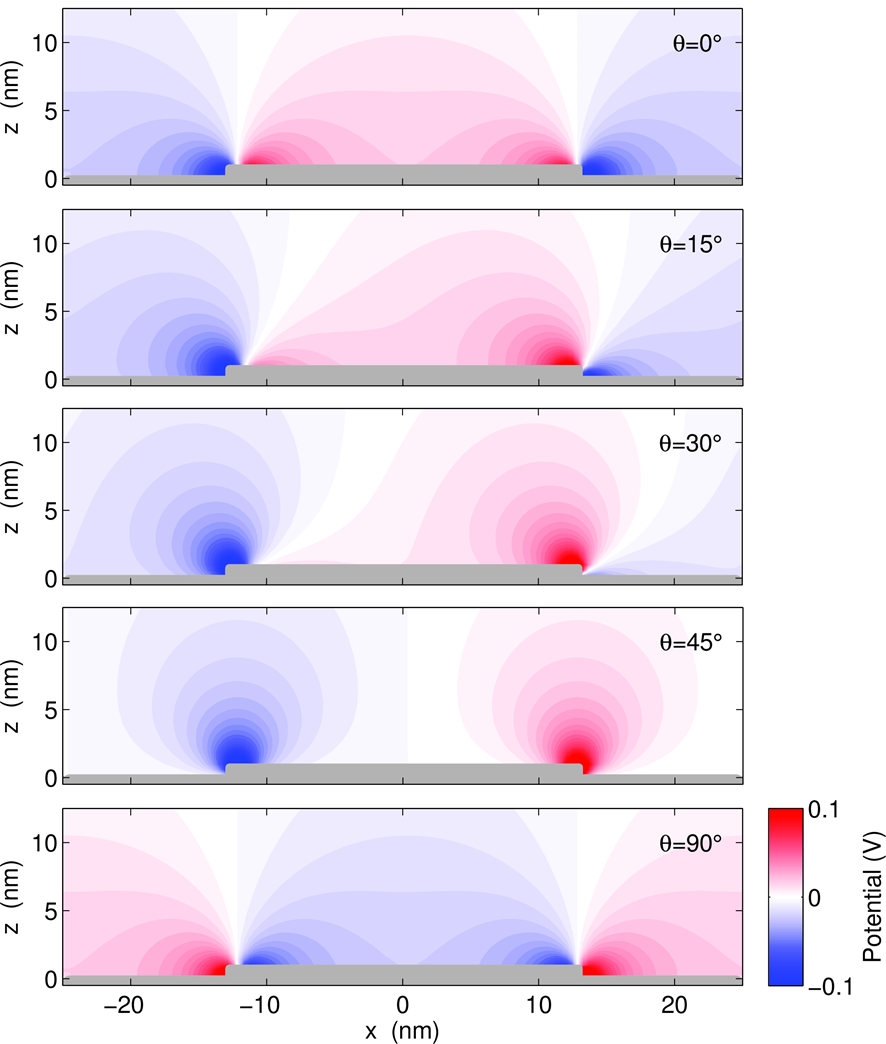


**Supplementary Figure 24 | Quadrupole tilt angle dependence of the electrostatic potential.** The rotation of molecular quadrupoles has an effect that is analogous to the rotation of step edge dipoles.

The effect of the tilt angle *θ* is addressed in Supplementary Figure 24. This angle can be both interpreted as physical tilting of a given molecule, but also as changes in the quadrupole layout due to the presence of electro-active substituents. The change in *θ* reflects an effective rotation of the step edge dipoles, with the sign of the potential that gets reversed upon rotation of 90°. The results for the point quadrupole model provide a qualitative rationalization of the behavior observed for Cl_2_-NDI, C_6_-NDI and PDIF-CN_2_, whose quadrupoles differ in magnitude and orientation. A precise mapping to the point-quadrupole model is however hindered by the complexity of real systems, where the angle *θ* is insufficient to characterize molecular orientations and the point quadrupole represents only a rough approximation. Atomistic results in Supplementary Figures 21-23 do instead account for all these effects in defect-free structures.


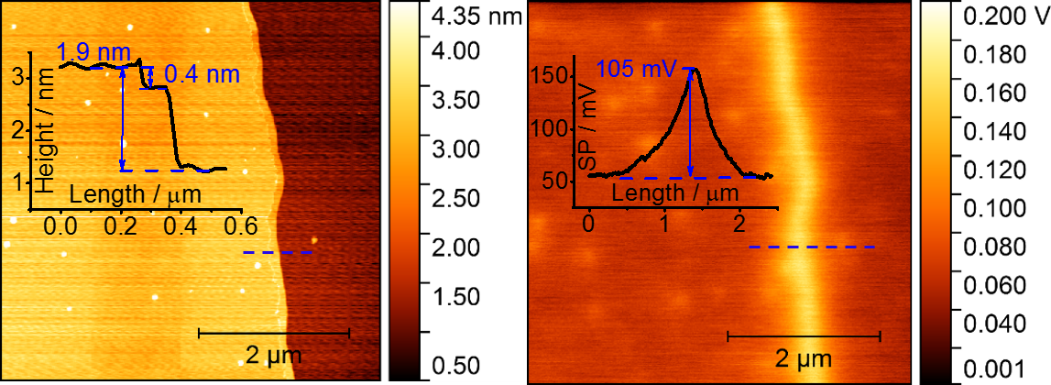


**Supplementary Figure 25 | AFM height (left) and surface potential images (right) for a Cl_2_-NDI single crystal stored in a glove box for over 6 months.** Insets show step height profile and corresponding surface potential profile along the dashed line. There is a clear 0.4 nm dip at the step edge that is not seen for freshly grown crystals and it may correspond to crystallographic reconstruction. The step edge potential has also increased during storage to ~+100 mV.


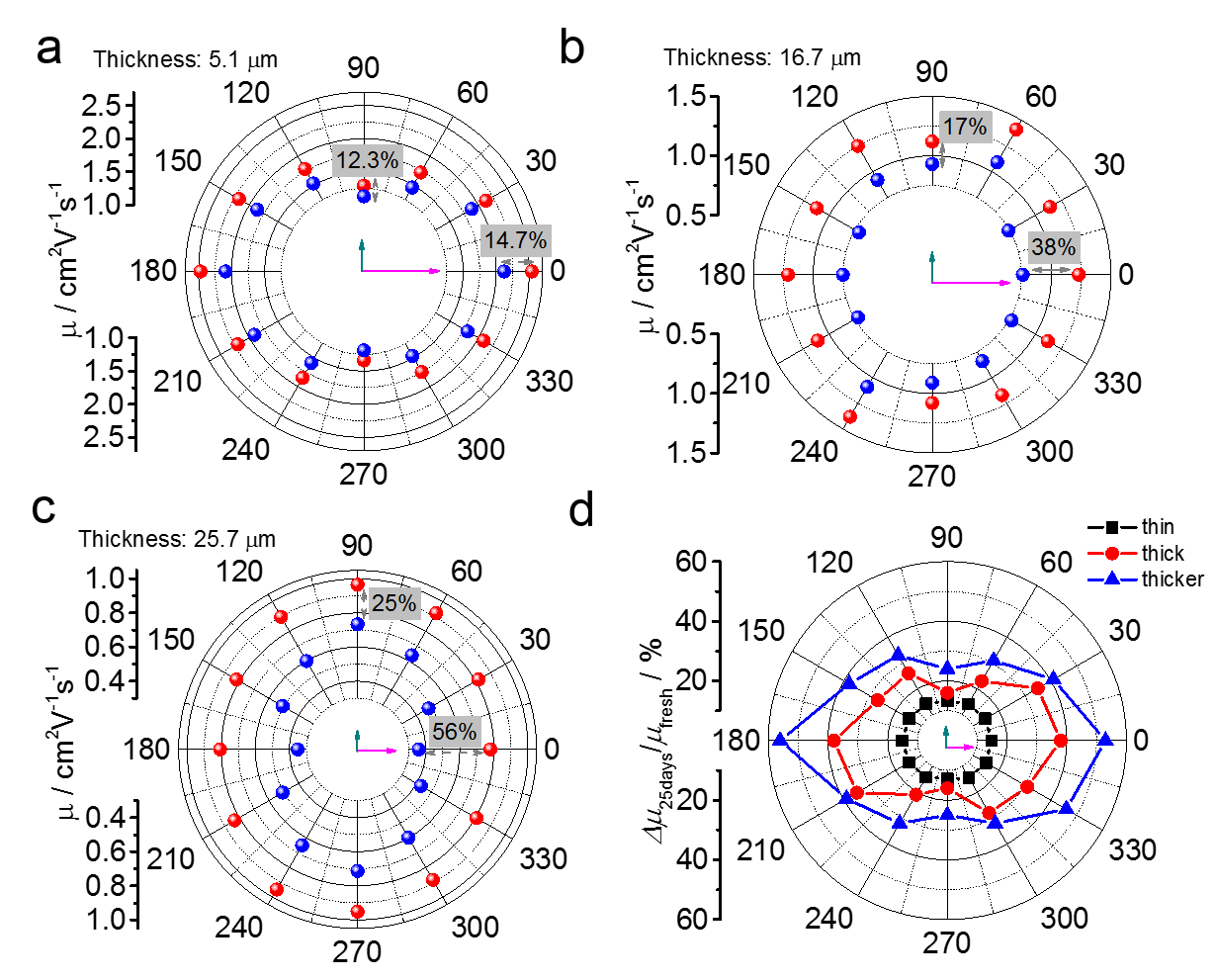


**Supplementary Figure 26 | The angular dependent device stability for Cl_2_-NDI single crystal with different thicknesses.** (**a**) thin single crystal (5.1 μm), (**b**) thick single crystal (16.7 μm) and (**c**) thicker single crystal (25.7 μm). Red and blue spheres represent mobility distributions for fresh single crystal and that stored in ambient for 25 days, respectively. Inserted percentages represent mobility degradation (∆*μ*_25days_/*μ*_fresh_) after 25 days along the respective direction. (**d**) Mobility degradation as a function of crystallographic directions. Almost isotropic mobility degradation is observed in thin single crystal FETs with the average value of 14%, perhaps owing to O_2_/H_2_O induced homogeneous defects and random distributed on the crystal surface. However, for thick/thicker single crystal FETs, significantly larger mobility degradation along 0° suggests that trap depth at step edge increases and deeper traps play a more crucial role in charge transport.


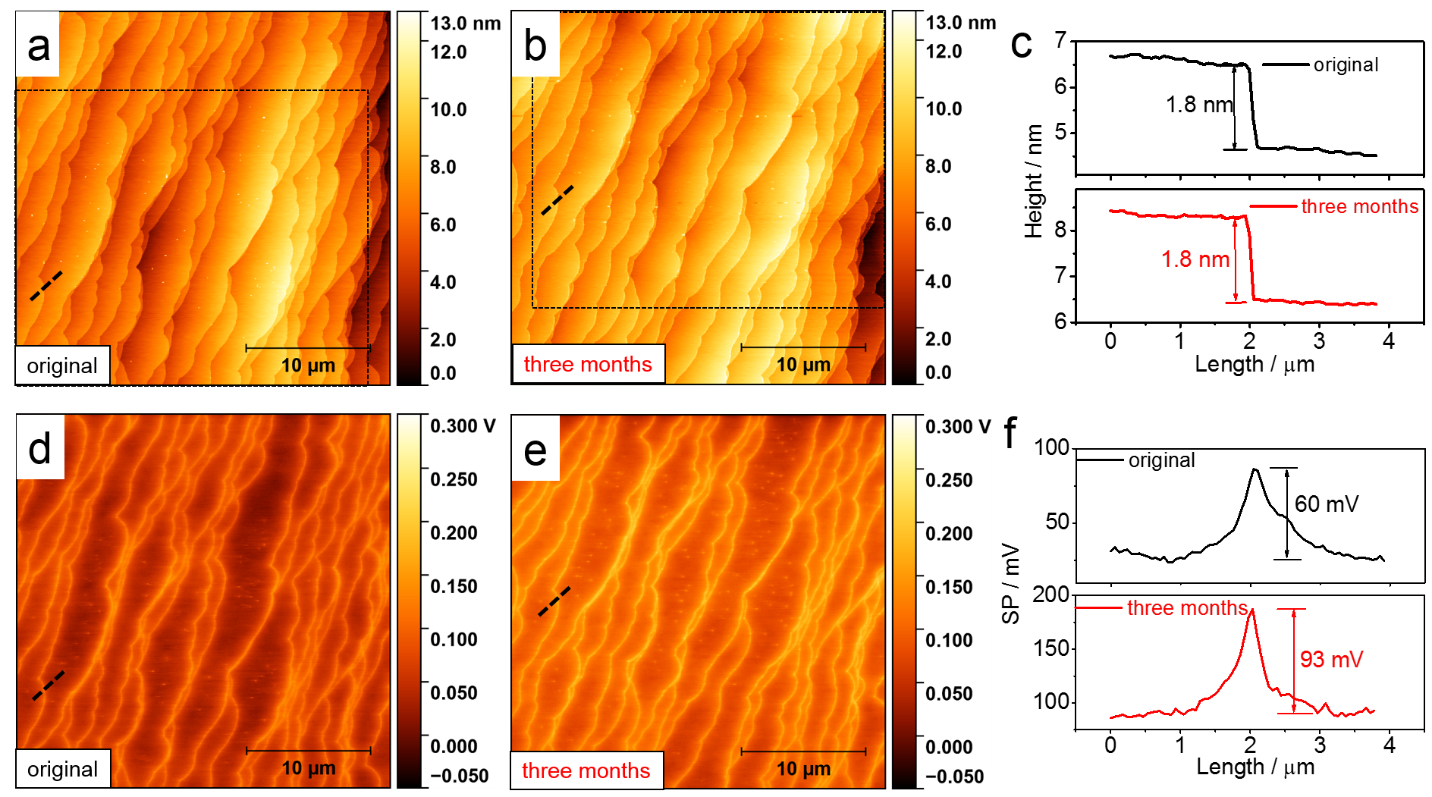


**Supplementary Figure 27 | In-situ AFM and SKPM scanning for a sample stored in ambient for three months.** AFM height images for (**a**) original single crystal and (**b**) the same crystal stored in ambient for three months. (**c**) Corresponding height profiles along dashed lines. The black dashed squares represent the same area. It shows the same topography after three months storage, which means no new steps were generated in ambient storage. SKPM surface potential images for (**d**) the original single crystal and (**e**) the same stored in ambient for three months. (**f**) Corresponding step edge potential profile along dashed lines. Step edge potential increases from 60 mV to ~ 90 mV.

**Supplementary Figure 28 | Step edge potentials as a function of the number of overlapping or intersecting molecular layers for fresh Cl_2_-NDI crystals and those aged 6 month in ambient.** Surface potential increases by 50% for samples stored in ambient for six months, which may be attributed to molecular orientation changes (restructuring) and oxygen related structural defects.

**Supplementary Figure 29 | Step edge surface potential changes for Cl_2_-NDI crystals upon annealing in vacuum and water vapor at 80 °C.** Six samples were placed in two Petri dishes (15 mm × 100 mm) and then sealed with parafilm, respectively. Both were kept in a vacuum of 0.1 torr and one petri dish was filled with 1 mL water. Samples were blown dry with N_2_ before SKPM measurement.


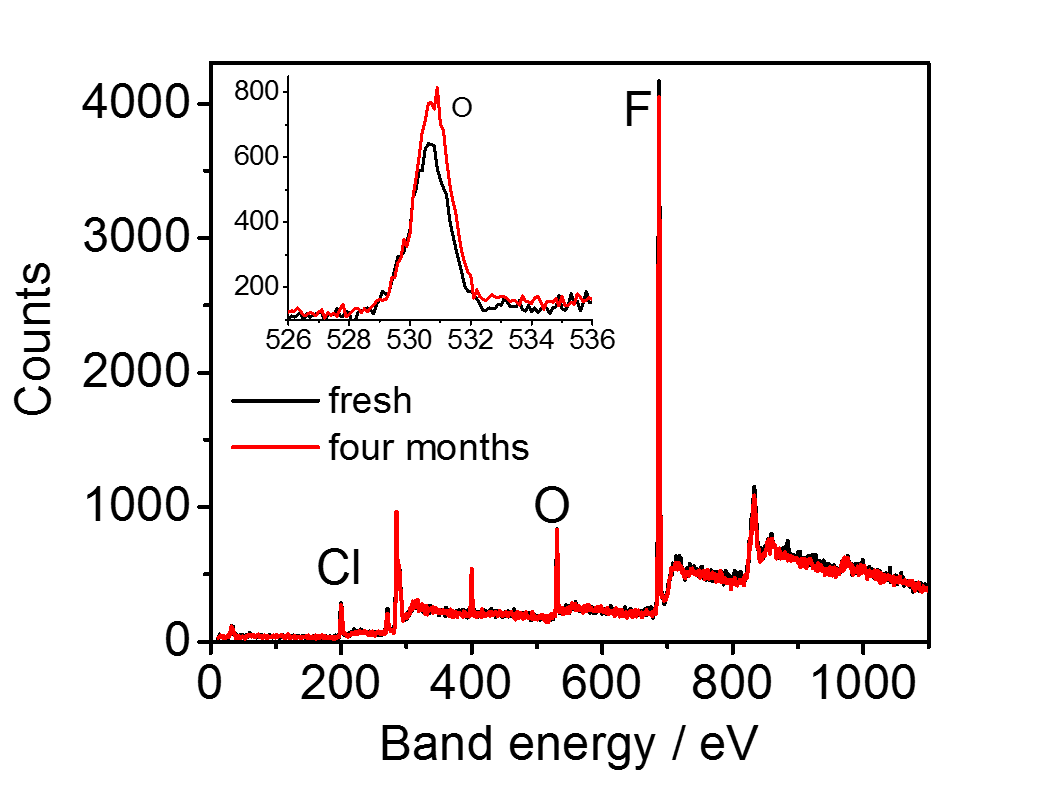


**Supplementary Figure 30 | X-ray photoelectron spectroscopy (XPS) of a fresh Cl_2_-NDI crystal and a crystal stored in ambient for four months.** Compared with the fresh crystals, the F_1s_ and Cl_1s_ peaks exhibit a slightly weaker intensity for the crystal stored in ambient for four months. The O_1s_ peak, however, shows a higher intensity and is wider, which may mean water and oxygen penetrate into the surface of the crystal. The area integrals of the F_1s_, Cl_1s_, O_1s_ peaks are 411.33, 42.14, and 71.64 for the fresh crystal and 390.45, 40.66, and 75.21 for the crystal stored in air for four months, respectively.

**Supplementary References:**

1. He, T. et al. Single-crystal field-effect transistors of new Cl_2_-NDI polymorph processed by sublimation in air. *Nat. Commun.* **6**, 5954 (2015).

2. Jones, B. A. et al. High-Mobility Air-Stable n-Type Semiconductors with Processing Versatility: Dicyanoperylene-3,4:9,10-bis(dicarboximides). *Angew. Chem. Inter. Edit.* **43**, 6363-6366 (2004).

3. Molinari, A. S., Alves, H., Chen, Z., Facchetti, A. & Morpurgo, A. F. High Electron Mobility in Vacuum and Ambient for PDIF-CN_2_ Single-Crystal Transistors. *J. Am. Chem. Soc.* **131**, 2462-2463 (2009).

4. Krupskaya, Y., Gibertini, M., Marzari, N. & Morpurgo, A. F. Band-Like Electron Transport with Record-High Mobility in the TCNQ Family. *Adv. Mater.* **27**, 2453-2458, (2015).

5. Minato, T. *et al*. High-resolution molecular images of rubrene single crystals obtained by frequency modulation atomic force microscopy. *App. Phys. Lett.* **95**, 093302, (2009).

6. Duhm, S. *et al.* Orientation-dependent ionization energies and interface dipoles in ordered molecular assemblies. *Nat. Mater.* **7**, 326-332, (2008).

7. D’Avino, G. *et al.* Electrostatic phenomena in organic semiconductors: fundamentals and implications for photovoltaics. *J. Phys.: Condens. Matter* **28**, 433002, (2016).

8. Besler, B. H., Merz, K. M. & Kollman, P. A. Atomic charges derived from semiempirical methods. *J. Comput. Chem.* **11**, 431-439, (1990).

9. Frisch, M. J., Trucks, G. W., Schlegel, H. B., Scuseria, G. E., Robb, M. A., Cheeseman, J. R., Scalmani, G., Barone, V., Mennucci, B., Petersson, G. A., Nakatsuji, H., Caricato, M., Li, X., Hratchian, H. P., Izmaylov, A. F., Bloino, J., Zheng, G., Sonnenberg, J. L., Hada, M., Ehara, M., Toyota, K., Fukuda, R., Hasegawa, J., Ishida, M., Nakajima, T., Honda, Y., Kitao, O., Nakai, H., Vreven, T., Montgomery Jr., J.A., Peralta, J. E., Ogliaro, F., Bearpark, M., Heyd, J. J., Brothers, E., Kudin, K. N., Staroverov, V. N., Kobayashi, R., Normand, J., Raghavachari, K., Rendell, A., Burant, J. C., Iyengar, S. S., Tomasi, J., Cossi, M., Rega, N., Millam, J. M., Klene, M., Knox, J. E., Cross, J. B., Bakken, V., Adamo, C., Jaramillo, J., Gomperts, R., Stratmann, R. E., Yazyev, O., Austin, A. J., Cammi, R., Pomelli, C., Ochterski, J. W., Martin, R. L., Morokuma, K., Zakrzewski, V. G., Voth, G. A., Salvador, P., Dannenberg, J. J., Dapprich, S., Daniels, A. D., Farkas, O., Foresman, J. B., Ortiz, J. V., Cioslowski, J. and Fox, D. J. Gaussian 09, Revision A.01, Gaussian, Inc., Wallingford CT, (2009).

10. Shukla, D. et al. Thin-Film Morphology Control in Naphthalene-Diimide-Based Semiconductors: High Mobility n-Type Semiconductor for Organic Thin-Film Transistors. *Chem. Mater.* **20**, 7486-7491, (2008).

11. Parry, D. E. The electrostatic potential in the surface region of an ionic crystal. *Surf. Sci.*, **49**, 433-440, (1975).

12. Gale, J. D. & Rohl, A. L. The General Utility Lattice Program (GULP). *Molecular Simulation* **29**, 291-341, (2003).

13. Řezáč, J. & Hobza, P. Advanced Corrections of Hydrogen Bonding and Dispersion for Semiempirical Quantum Mechanical Methods. *J. Chem. Theory Comput.* **8**, 141-151, (2012).

14. Stewart, James J. P. MOPAC2016, Stewart Computational Chemistry, Colorado Springs, CO, USA, [HTTP://OpenMOPAC.net], (2016).
